# Protein crowding and lipid complexity influence the nanoscale dynamic organization of ion channels in cell membranes

**DOI:** 10.1038/s41598-017-16865-6

**Published:** 2017-11-30

**Authors:** Anna L. Duncan, Tyler Reddy, Heidi Koldsø, Jean Hélie, Philip W. Fowler, Matthieu Chavent, Mark S. P. Sansom

**Affiliations:** 1 Department of Biochemistry, University of Oxford, Oxford, OX1 3QU UK; 2 Present Address: T-6, MS K710, Los Alamos National Laboratory, Los Alamos, NM 87545 USA; 3 Present Address: D. E. Shaw Research, 120 W 45th St., New York, NY 10036 USA; 4 Present Address: Semmle, Blue Boar Court, 9 Alfred St, Oxford, OX1 4EH UK; 5 Present Address: Nuffield Department of Medicine, University of Oxford, John Radcliffe Hospital, Oxford, OX3 9DU UK; 6 Present Address: IPBS-CNRS, Toulouse, Midi-Pyrénées France

## Abstract

Cell membranes are crowded and complex environments. To investigate the effect of protein-lipid interactions on dynamic organization in mammalian cell membranes, we have performed coarse-grained molecular dynamics simulations containing >100 copies of an inwardly rectifying potassium (Kir) channel which forms specific interactions with the regulatory lipid phosphatidylinositol 4,5-bisphosphate (PIP_2_). The tendency of protein molecules to cluster has the effect of organizing the membrane into dynamic compartments. At the same time, the diversity of lipids present has a marked effect on the clustering behavior of ion channels. Sub-diffusion of proteins and lipids is observed. Protein crowding alters the sub-diffusive behavior of proteins and lipids such as PIP_2_ which interact tightly with Kir channels. Protein crowding also affects bilayer properties, such as membrane undulations and bending rigidity, in a PIP_2_-dependent manner. This interplay between the diffusion and the dynamic organization of Kir channels may have important implications for channel function.

## Introduction

Cell membranes are crowded and complex environments, containing up to 50% protein by mass, which is equivalent to ca. 25% protein by cross-sectional area^1^. They contain a multiplicity of lipid species distributed asymmetrically between the outer and inner leaflets of the bilayer^2–4^. Due to the high area fraction of protein in cell membranes, protein crowding occurs. This has been implicated in a number of biological processes^5–7^, and protein nanoclustering has been shown to be an important factor in membrane organisation^8,9^. It has been shown that membrane proteins co-diffuse as complexes with lipids^10^, and that protein crowding affects both protein and lipid diffusion^11–13^.

There is a growing appreciation of the importance of specific protein-lipid interactions and their role in both regulating protein activity and contributing to the dynamic high order organization of cell membranes^14–16^. Molecular simulations in particular have proved a useful tool to study lipid interactions with membrane proteins^17–19^.

In addition to probing the nature of protein-lipid interactions, there has been much interest in how complex lipid mixtures may generate dynamic organization in lipid bilayers. This has embraced e.g. lateral nanodomains of specific lipid composition^20,21^, and also the contribution of lipid properties to membrane curvature^22^ and fluctuations^23^. Despite impressive progress^7^ much remains to be understood regarding how the molecular interactions between protein and lipids, including crowding, influence higher-level membrane organization and dynamics^24^. Advances in both coarse-grained (CG) force fields^25^ and in computational power mean that large-scale CG molecular dynamics (MD) simulations are increasingly being used to provide insights that can be directly linked to experiments^26,27^.

In order to investigate the role of protein crowding and lipid complexity on the organization and dynamics of a model membrane containing a protein whose activity is regulated by interactions with lipids, we have focused on the ion channel Kir2.2. This is a member of the inward rectifier family of potassium channels, which have well-characterized and specific lipid interactions^28–30^. Kir channels help to restore the electrical voltage across a membrane to its resting potential in excitable cells^31^. Kir2.2 channels are tetrameric proteins containing a classical potassium channel transmembrane domain coupled to a large cytoplasmic domain^32^. Kir channels are activated by phosphatidylinositol 4,5-bisphosphate (PIP_2_), an anionic lipid present in the inner leaflet of mammalian plasma membranes. PIP_2_ binds to four identical interaction sites on the surface of the tetrameric channel protein^32,33^. Secondary anionic lipid species (e.g. phosphatidyl serine) may further modulate the activity of the channel, and an additional anionic interaction site on each subunit has been identified^29,34,35^. Additionally, cholesterol inhibits the channel *via* direct protein-lipid interactions^36,37^. The cytoplasmic domain of the homologous Kir2.1 channel has been shown to form clusters in the presence of channel-organising protein, PSD-95 (see below)^38^.

There is mounting biophysical evidence for ion channel clustering in cell membranes, and for its functional importance e.g.^39^. Clustering of the canonical bacterial potassium channel KcsA is influenced by anionic lipids^40^, with the channel forming large salt-induced complexes^41^. In reconstituted membranes KcsA can form extended clusters up to a micrometer in size which are observable by confocal microscopy^42^. Other bacterial channels have also been demonstrated to cluster, for example including the mechanosensitive channel MscL^43^. Several studies have implicated lipids in channel clustering mechanisms, e.g. lipid microdomains were identified as being important in Ca_v_2.1 clustering^44^. Early functional studies of A-type potassium channels suggested the formation of clusters of ~50 channels^45^, and fluorescence imaging studies revealed that L-type Ca^2+^ channels form clusters of ~100 nm diameter^46^. The canonical MAGuK (membrane-associated guanylate kinase) scaffold protein PSD-95 aids the formation of potassium channel clusters on postsynaptic membranes^47,48^. A number of studies have indicated that Kir channels interact with PSD-95^49–53^ and the isolated cytoplasmic domain of Kir2.1 has been shown to form clusters on association with PSD-95^38^. It is therefore timely to examine the extent to which Kir channels cluster when embedded in model membranes at a surface density comparable to that in mammalian cell membranes^1^, and how the resultant dynamic organization of channel molecules may be influenced by the lipid environment.

Here we present large-scale CG molecular dynamics simulations of Kir2.2 channels embedded in a range of membranes of varying degrees of lipid complexity and protein crowding. We dissect the interplay of protein crowding and lipid dynamics, and show that the interactions between Kir channels and PIP_2_ play a key role in the dynamic organization of a biologically realistic model of cell membranes.

## Results and Discussion

### Simulations of Crowded Kir-Containing Membranes

**Figure 1:**
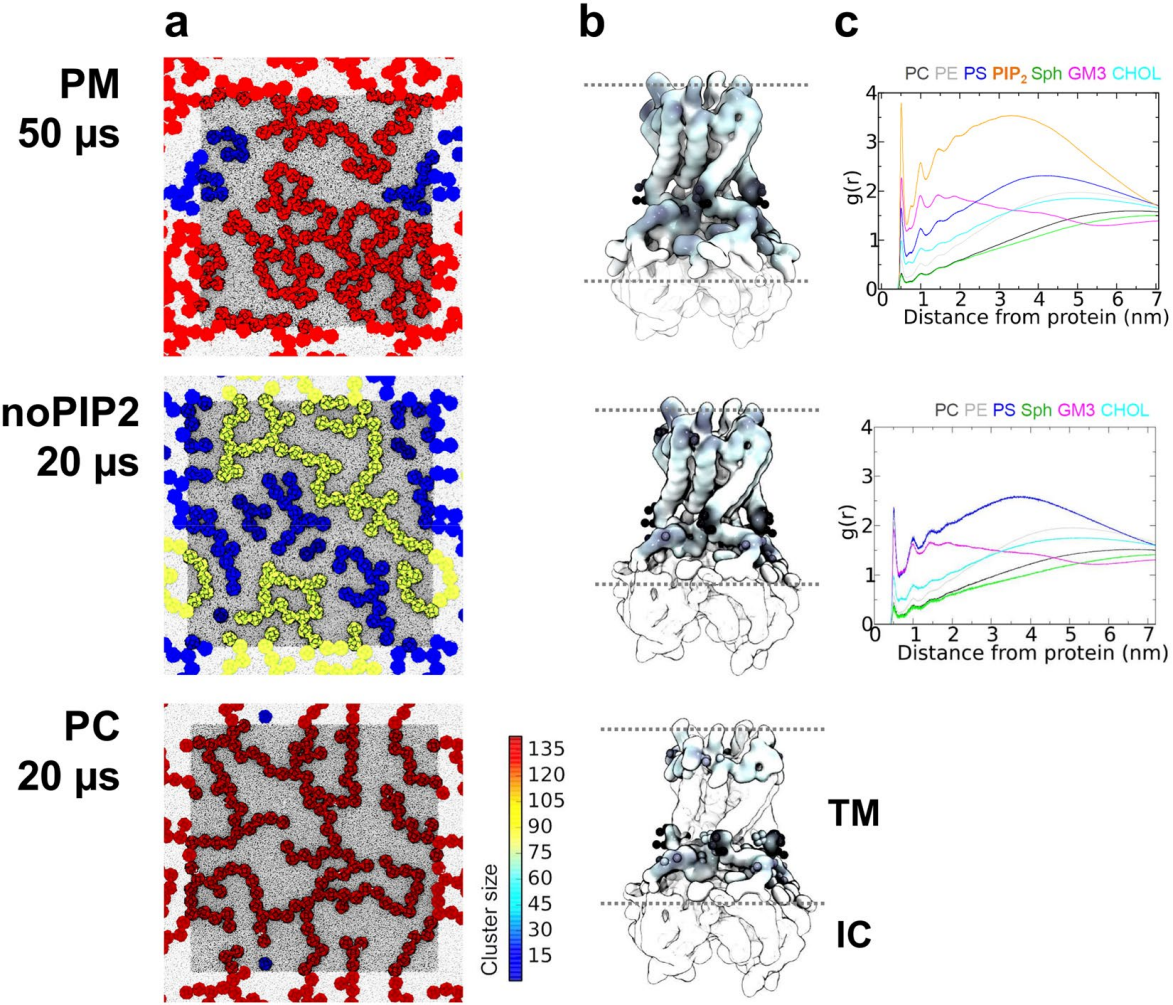
Simulations of Kir2.2 in complex lipid bilayers. (**a**) Final snapshots of the PM, noPIP2, and PC simulations (see Table 1 for details). The membrane is viewed from the inner (cytoplasmic) side, with proteins colored according to the cluster size, and lipids colored dark grey. The approximate simulation box size (shown in grey) is 137 × 137 nm^2^. (**b**) The Kir2.2. structure with lipid headgroup interactions mapped onto the channel surface using a greyscale from white (no interactions) to grey (many interactions). Residues side chains are shown for those residues at which lipid headgroup interactions account for more than 2.5% of the total interactions each. The approximate location of the lipid bilayer is indicated by two broken lines on each structure, with the transmembrane (TM) and intracellular (IC) domains indicated on the diagram corresponding to the PC simulation. (**c**) Radial distribution functions of the distribution of lipid headgroups around Kir2.2 channels for the PM and noPIP2 simulations.

The simulations performed are summarized in Table 1 (see also Supplementary Table S1). To explore the dynamic organization of Kir channels we simulated 144 copies of the Kir2.2 channel embedded in a plasma membrane (PM) model. This provides a membrane area (of 137 × 137 nm^2^) with a sufficient density of proteins for clustering to be observed. The model was simulated for 50 μs to allow sufficient time for the protein and lipid dynamics to be extensively explored (Fig. 1a). The bilayer of the PM model contained the major lipid species observed in a mammalian plasma membrane^54^. This model has previously been used to explore GPCR clustering^55^. In order to explore the effect of PIP_2_–Kir channel interactions on the behavior of the ion channel, a 20 μs simulation of 144 Kir2.2 channels in a control plasma membrane lacking PIP_2_ was performed (noPIP2; Table 1). Finally, to explore the effects of lipid complexity, an additional control simulation of 144 Kir2.2 channels in a simple PC membrane was performed for 20 μs.

**Table 1:** Simulations Performed.

simulation	number of proteins	number of lipids	bilayer composition*	box length (nm)	duration (µs)
*Large Systems (~55,000 lipids; ~3.5* *M particles)*					
PM	144	55,584	PM	137	50
noPIP2	144	55,584	PM – PIP2	130	20
PC	144	55,728	PC	145	20
PMsparse	36	63,252	PM	131	20
PMnoprot	0	54,000	PM	120	10
PCnoprot	0	54,684	PC	136	5
*Small Systems* (~*3500 lipids*; ~*0.2* *M particles*)					
PM_s	9	3,474	PM	34	20
noPIP2_s	9	3,474	PM – PIP2	33	20
noG_s	9	3,483	PM – GM3	32	20
noPIP2G_s	9	3,474	PM -PIP2 – GM3	33	20
noCh_s	9	3,474	PM - Chol	35	20
noP2GCh_s	9	3,474	PM – PIP2 – GM3 - Chol	35	20
PC_s	9	3,483	PC	36	20

*PM = inner: PC:PE:PS:PIP2:Chol (10:40:15:10:25) & outer: PC:PE:Sph:GM3:Chol (40:10:15:10:25); for the detailed compositions of other bilayers see SI.

A summary of lipid headgroup interactions with Kir channels observed in the simulations is given in Fig. 1b. In the simulation containing PIP_2_, headgroup interactions with Kir2.2 channels extended further from the transmembrane domain onto the cytoplasmic domain of the channel (comparing top and middle panels in Fig. 1b). The radial distribution functions (Fig. 1c) indicate that in the PM simulation, the principal lipids surrounding Kir2.2 channels are PIP_2_ in the cytoplasmic leaflet and GM3 in the outer leaflet. PS was the second most populous inner leaflet lipid in the annular shell around Kir2.2 channels. In noPIP2 simulations, the abundance of PS at the protein surface was increased, but not to the extent for PIP_2_ in the PM simulations. Thus it can be seen that the major interactions of the protein are with PIP_2_ in the inner leaflet, and with GM3 in the outer leaflet, as has been shown to be the case for the EGF receptor^56^.

### Lipid Complexity and Dynamic Channel Protein Organization

**Figure 2:**
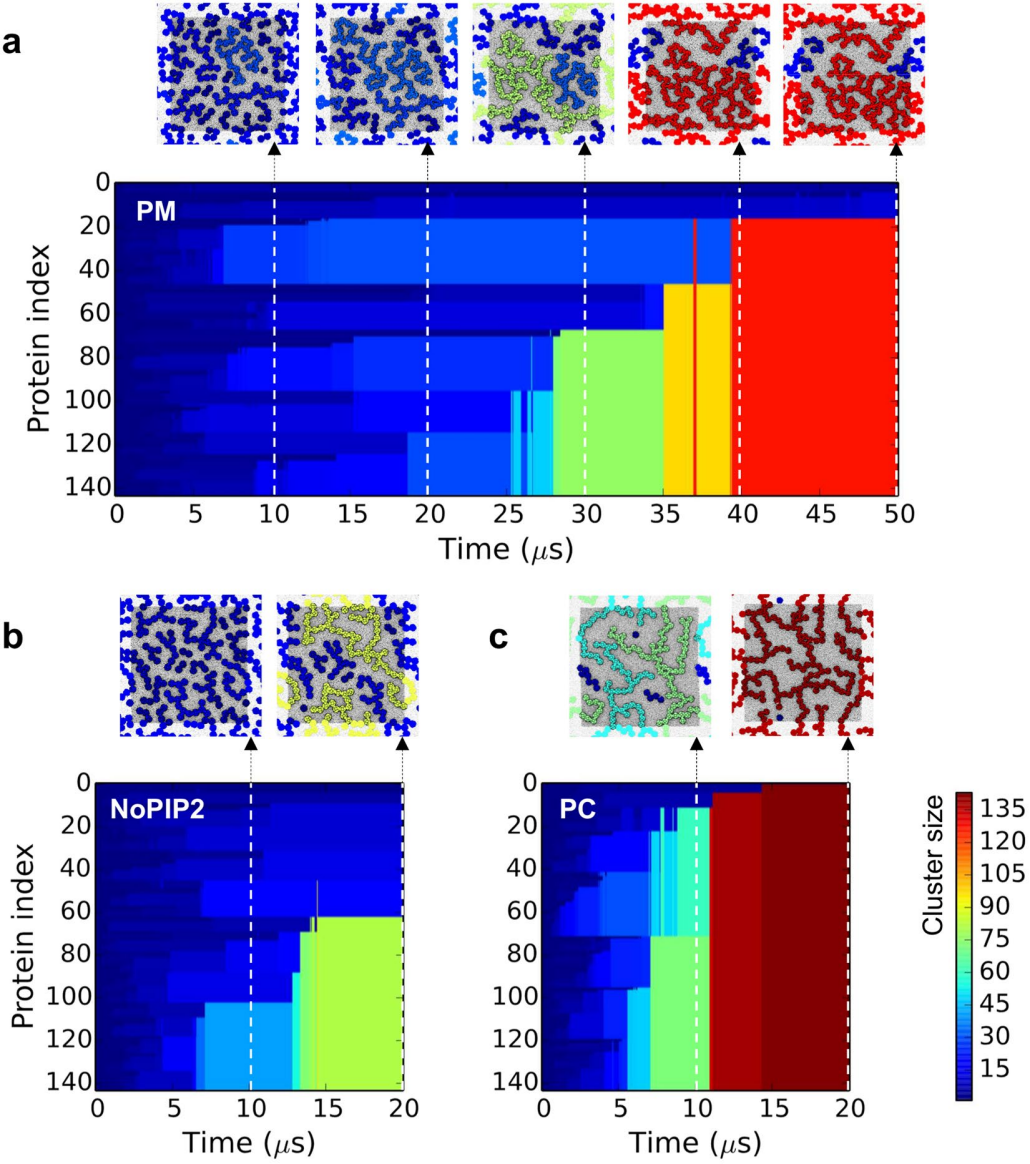
Evolution of Kir clusters over simulation time for the (**a**) PM, (**b**) noPIP2 and (**c**) PC simulations. A color code is used to indicate the cluster size ranging from deep blue (single channels) to deep red (clusters of >135 channels). Above each simulation snapshots of the membrane are shown every 10 μs, in the same format as in Fig. 1a.

**Figure 3:**
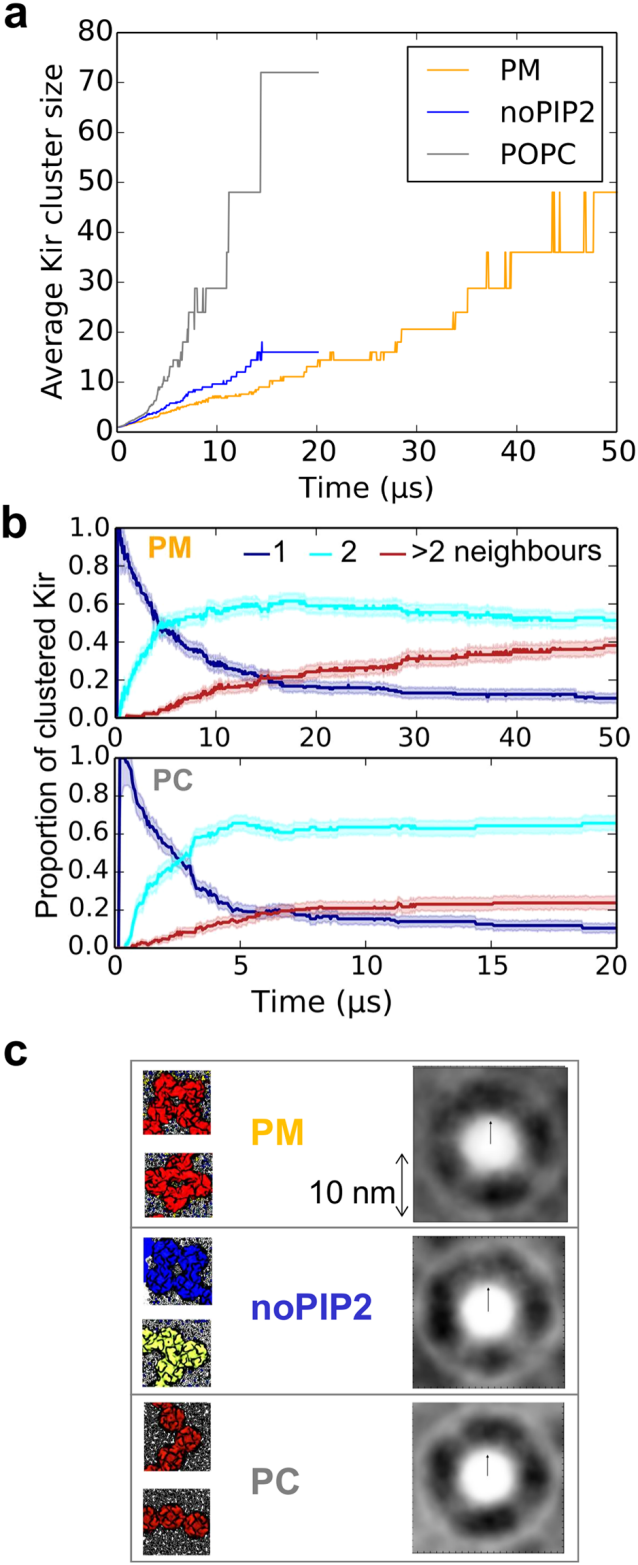
Cluster size and shape as functions of simulation time. (**a**) Averag cluster size over the course of each simulation. (**b**) Proportion of the total number of channel proteins with 1, 2 or >2 protein neighbours over the course of each simulation for the PM (top panel) and PC (lower panel) simulations. Errors are the bootstrap estimate of the standard error, derived by resampling the data with replacement using 1000 iterations. (**c**) On the left, zoomed in views of groups of 4 (PM), 4 and 3 (noPIP2), and 3 (PC) Kir channels selected from the final simulation snapshots shown in Fig. 1a. On the right are shown distributions of Kir channel pairs (on a greyscale of relative frequency from white = 0 to black = 1), derived from the last microsecond of each 50 µs (PM) or 20 µs (noPIP2 and PC) simulation.

The organization of Kir2.2 channels after 20–50 μs of simulation in membranes of differing lipid complexity are illustrated in Fig. 1a. Substantial differences in the clustering of Kir2.2 channels are apparent. In membranes with complex lipid mixtures (PM and noPIP2), the ion channel clusters are smaller and appear to be more branched (especially in the PM simulation). If we track cluster formation (Fig. 2) and cluster size (Fig. 3a) as functions of time this difference is especially evident. After 15 µs most of the channel proteins in the PC membrane form a single cluster containing ca. 120 proteins (Fig. 2c) whereas at the same time in the PM simulation clusters range up to ca. 30 proteins in size (Fig. 2a), with the noPIP2 simulation exhibiting intermediate behavior (Fig. 2b).

It is evident from the average cluster size *vs*. time that Kir clusters form more rapidly in the PC membrane (Fig. 3a). There is also a noticeable difference in cluster shape between the different bilayer compositions. Clusters in the more complex lipid mixtures are less linear in shape than in the PC membrane. Cluster shape can be characterized by the number of neighbors each protein has: clusters with a linear shape typically contain proteins with only two neighbors, whereas clusters that are more branched contain a high fraction of proteins with more than two neighbors (Fig. 3b and c). Cluster shapes can be compared by looking at the largest cluster formed of ~100 channels, occurring after 40 μs in PM and 20 μs in the PC simulations. The proportion of proteins with only one neighbor is similar in both PM and PC simulations, over the timescale that the largest cluster is formed. However, the proportion of proteins with two or more than two neighbors appear to diverge: in the PM simulations, 35–40% of proteins have three or more neighbors, whereas in the PC simulations only ~25% have three or more neighbors.

**Figure 4:**
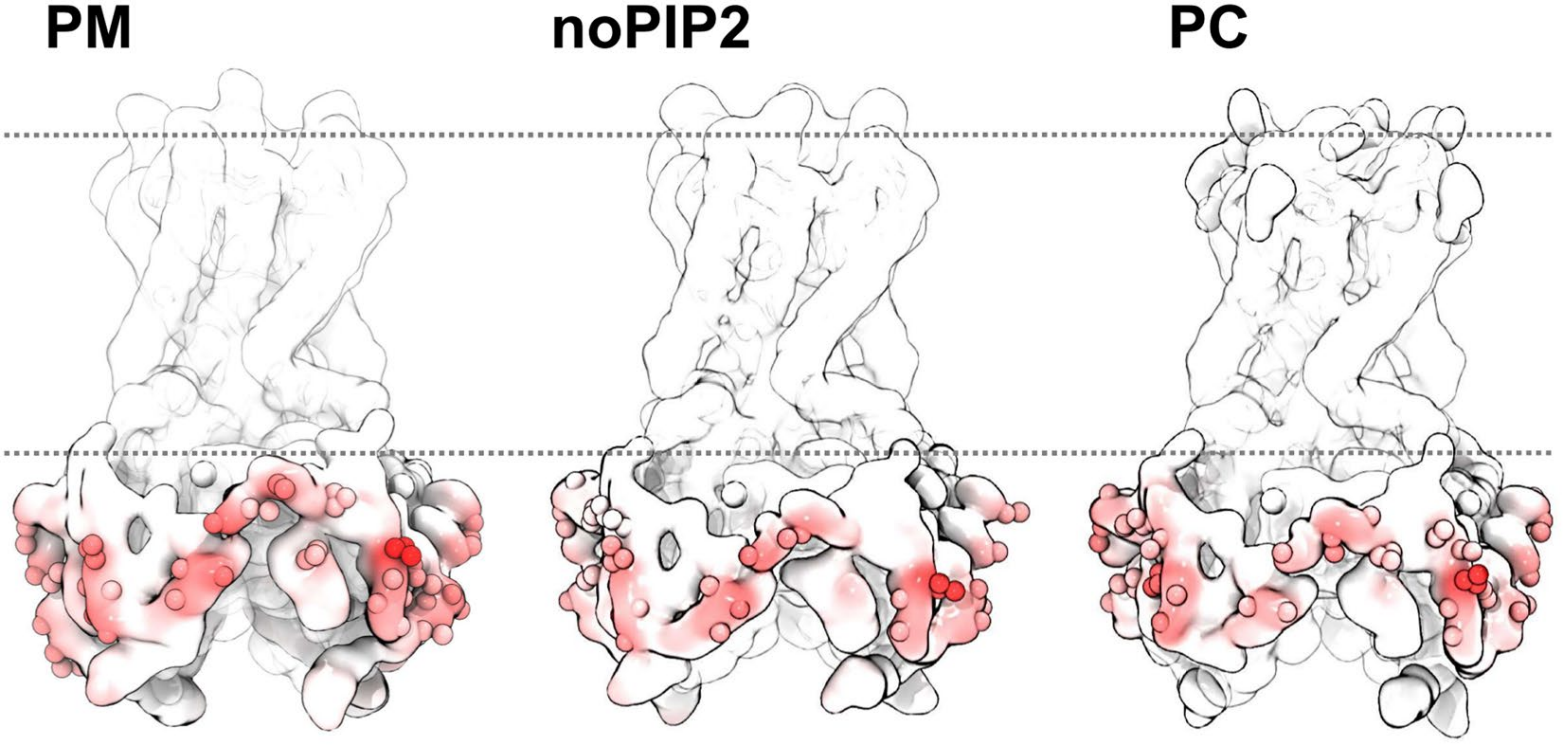
Residues involved in protein-protein interactions. Residues which interact with high frequency (i.e. over 0.7% of total interactions) with a neighboring Kir channel are shown as spheres. Interaction frequency is shown as a heat map from white (little interaction) to red.

What underlies this difference in organization? The frequency and pattern of protein-protein interactions, as estimated via the spatial frequency of pairwise interactions (Fig. 3c) and the map of interacting residues (Fig. 4), are similar in all three simulations and are largely mediated by the intracellular domain of Kir. Furthermore, even though Kir2.2 binds PIP_2_ molecules close to its intracellular domain (see Fig. 1c), the presence or absence of PIP_2_ does not seem to influence channel clustering. Therefore, the influence of lipids on the dynamic organization and clustering of Kir channels does not appear to be mediated directly through protein-lipid interactions, but more likely is an effect of changes in lipid diffusion within the plasma membrane upon increasing lipid complexity.

### Lipid Diffusion Rates

**Figure 5:**
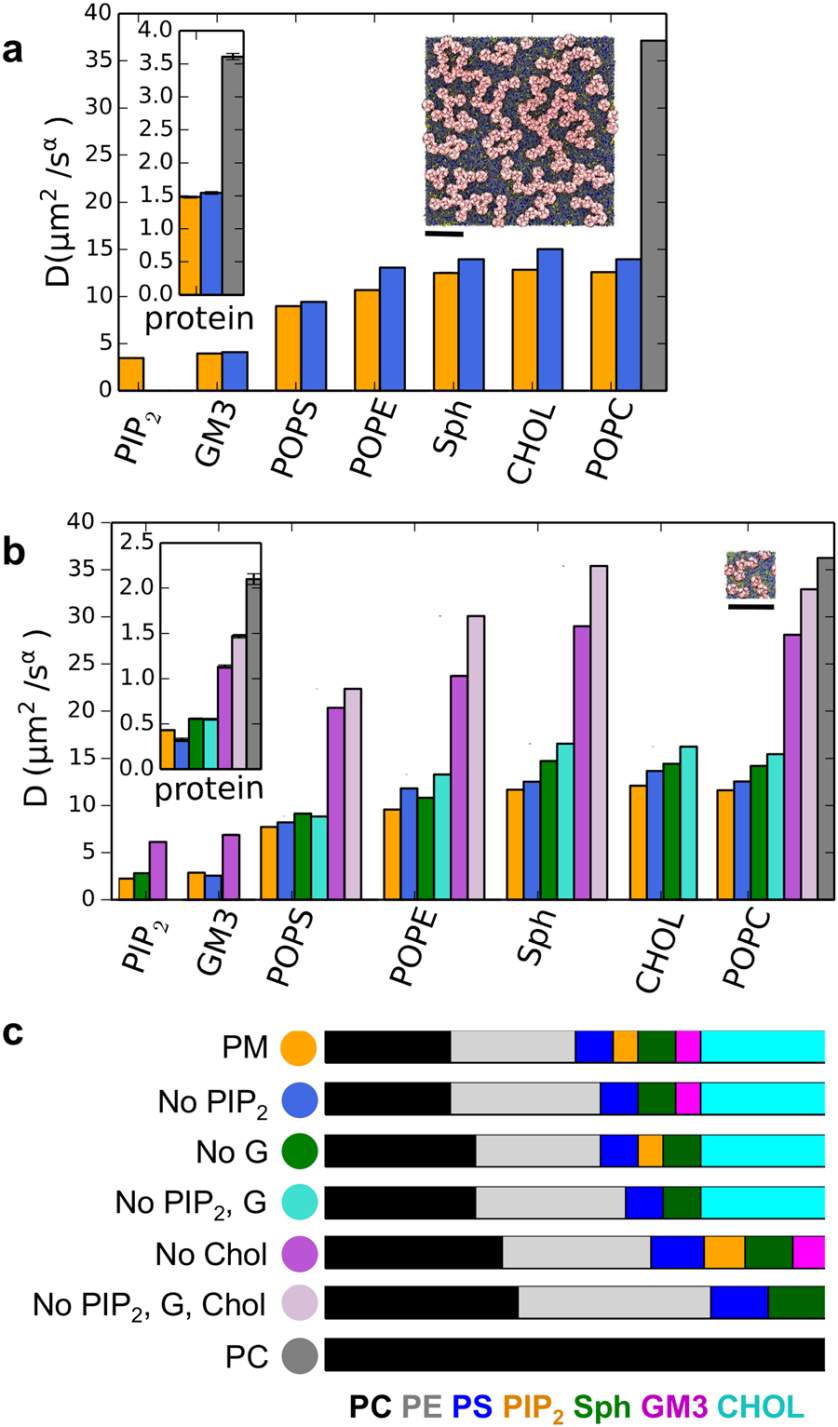
(**a**) Diffusion coefficient of each lipid species in the PM (orange-yellow bars); noPIP_2_ (blue bars); and in the PC (grey) simulations, with the Kir protein diffusion coefficients shown as an inset. A snapshot of the PM system at 20 μs is shown as an inset. The black scale bar shows 30 nm. Diffusion coefficients are also listed in Table S2. (**b**) Diffusion coefficients for the small simulations systems, again with protein diffusion coefficients as an inset (see Table 1 for details of system compositions, and Table S2 for diffusion coefficients listed). The small system size is indicated by the inset, as before with the black scale bar showing 30 nm. (**c**) Color codes for each simulation in A and B are shown along with lipid proportions for each simulation system, with lipid coloured: PC (black), PE (grey), PS (blue), PIP_2_ (orange-yellow), Sph (green), GM3 (magenta) and cholesterol (cyan).

The diffusion rates of the component lipids and proteins in the different membrane simulations appear to correlate with the clustering behavior of the channel proteins (Fig. 5a). Both the channel proteins and all lipid species in PM and noPIP2 simulations diffuse more slowly than in the PC simulation. For example, for the channel proteins the diffusion coefficients are ~2 *vs*. 4 μs^2^/s^α^ in PM/noPIP2 *vs*. PC respectively, and for PC molecules the diffusion coefficients are 12–14 μs^2^/s^α^ *vs*. 37 μs^2^/s^α^ for PM/noPIP2 *vs*. PC simulations respectively. This in turn suggests that the difference in rates and pattern (linear vs. branched) of cluster formation might reflect the diffusional behavior of lipids and proteins in the bilayers.

To investigate which lipid species is responsible for the reduction in diffusion, further simulations were performed for a variety of complex lipid membranes. These were scaled down in size to yield a system with 9 Kir2.2 channels embedded in a smaller membrane, whilst maintaining the same protein:lipid ratio as for the larger systems (Tables 1 and S1). In order to understand the effect of specific protein-lipid interactions on diffusion, simulations of this smaller system were run for a plasma membrane (PM) model, and for a PM with PIP_2_ removed, or GM3 removed, or with both of these lipid species removed. To assess the contribution of cholesterol (Chol) to membrane dynamics, simulations of 9 Kir2.2 channels in a PM with cholesterol removed, or with PIP_2_, GM3 and Chol all removed were also run (see Table 1 for details).

The effect of removing the lipid species which interact with the protein (that is, PIP_2_ and GM3 – see above) is to increase the diffusion coefficient slightly for all lipid species (Fig. 5b and Table S2). Removal of either PIP_2_ or GM3 mainly alters the diffusion coefficients of the lipids in the same leaflet, (i.e. in the inner or outer leaflet respectively). It is notable that this trend does not hold for PS; even though PS and PIP_2_ are both in the inner leaflet of the plasma membrane, on removal of PIP_2_, the diffusion coefficient of PS does not increase as much as that of e.g. PE. This may be because PS retains a high level of interaction with Kir channels. Cholesterol is present at equal levels in both leaflets. Its diffusion coefficient was affected slightly more by the removal of GM3 than that of PIP_2_. Removal of both PIP_2_ and GM3 caused a slightly greater increase in the diffusion coefficients of all remaining lipid species than when PIP_2_ or GM3 were removed individually, and the effect appeared to be additive, for example POPE diffusion was 10 μs^2^/s^α^ in the PM_s simulation, increasing to 12 μs^2^/s^α^ on removal of PIP_2_ (noPIP2_s simulation), and to 11 μs^2^/s^α^ on removal of GM3 (NoG_s simulation). After removal of GM3 and PIP_2_ (NoPIP2G_s simulation), POPE had a diffusion coefficient of 13 μs^2^/s^α^. Similar trends were observed for sphingomyelin, cholesterol and POPC.

The effect of cholesterol on membrane diffusion has been much investigated and has been shown to slow diffusion (see e.g.^57–59^). As a control, we removed cholesterol, both as the sole species taken out of the plasma membrane and concurrently with the removal of PIP_2_ and GM3. Whereas removal of PIP_2_ and/or GM3 caused little increase in the diffusion coefficient of the Kir channels and the remaining lipid species (the maximum increase on removal of PIP_2_ and GM3 was for sphingomyelin (Sph) which increased by 5 μs^2^/s^α^ from PM_s to noP2G_s) in the membrane, removal of cholesterol caused a much greater increase in the diffusion coefficient of all remaining lipid species. Removal of cholesterol from the plasma membrane has a particularly marked effect on sphingomyelin diffusion, as might be anticipated^60^ (although the nature of the cholesterol-sphingolipid interaction in living cell membranes is currently the topic of much debate^61^). Moreover, while the removal of PIP_2_ and/or GM3 had little effect on the diffusion coefficient of Kir2.2 channels (an increase from 0.4 μs^2^/s^α^ in PM_s to 0.6 μs^2^/s^α^ in noP2G_s), removal of cholesterol almost tripled the protein diffusion coefficient (from 0.4 μs^2^/s^α^ in PM_s to 1.1 μs^2^/s^α^ in noChol_s). However, even with the removal of much of the lipid complexity, protein and lipid diffusion is not increased to the levels observed in the single-component PC membrane. Thus, diffusion of the lipid PC is 3 μs^2^/s^α^ lower in PM simulations (comprised of a lipid mixture containing at least PC, PE, PS and sphingomyelin) than in PC simulations (which contain only PC).

### Protein Crowding Decreases Diffusion Coefficient

**Figure 6:**
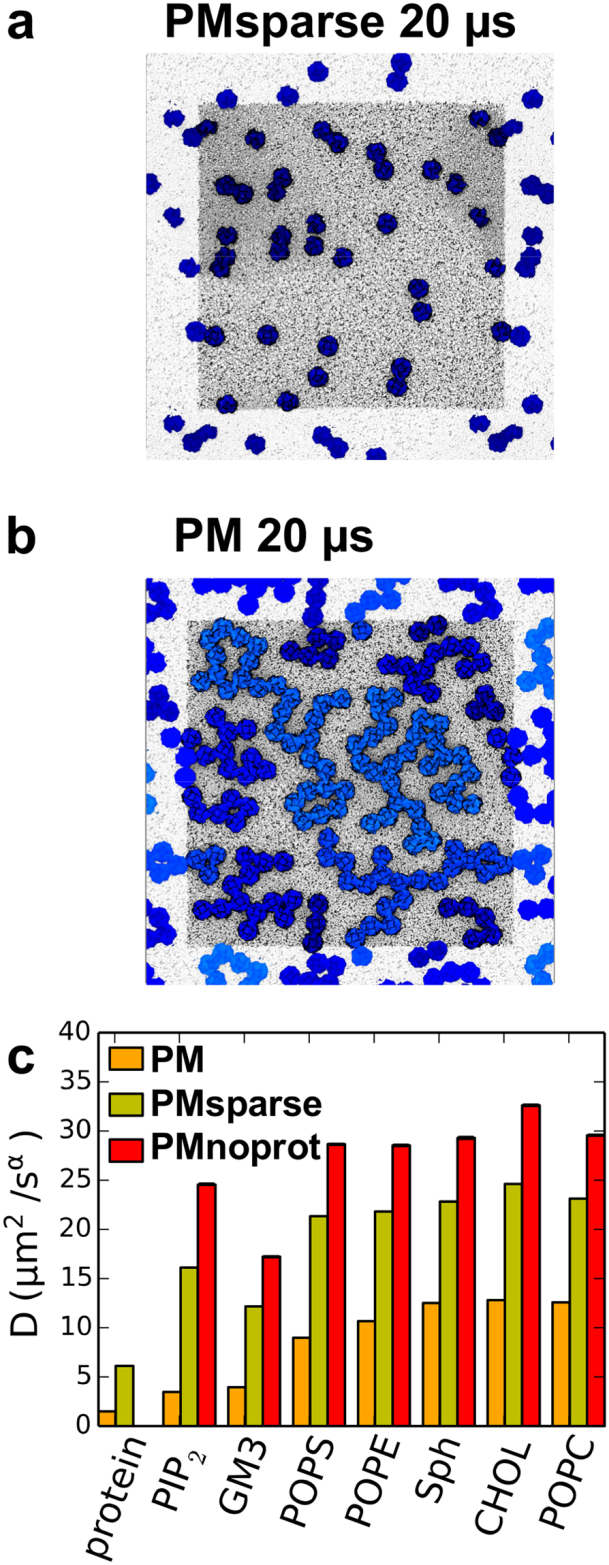
Snapshots of the (**a**) uncrowded PMsparse and (**b**) crowded PM systems, both at 20 μs, with proteins colored according to cluster size (as in Fig. 1a), viewed from the cytoplasmic side. (**c**) Effect of crowding on diffusion, shown as diffusion coefficients of proteins and each lipid species in the crowded PM (orange-yellow), the uncrowded PMsparse (lime green) and the PMnoprot (red) simulations. Diffusion coefficients are also listed in Table S2.

In order to investigate the effect of protein crowding on diffusion and channel clustering, a simulation was performed in which only 36 Kir channels were embedded in a 120 × 120 nm^2^ membrane, thus giving a system (PMsparse; Table 1) with 25% of the protein density of the PM simulations (Fig. 6). Protein clusters in this sparsely populated membrane were much smaller, with typically at most dimers of Kir channels formed on the timescales simulated here. As a further control, a simulation of the plasma membrane containing no protein (PMnoprot) was also performed. Decreasing protein crowding caused both protein and lipid diffusion coefficients to increase significantly, although not quite to the values for the PMnoprot system. Thus, reducing the protein density to the extent that crowding appeared to no longer play a role (PMsparse) increased lipid diffusion coefficients to values of ~60% of the PMnoprot coefficients (Fig. 6c). A further control simulation of a PC membrane containing no protein (PCnoprot) also showed lipid diffusion to increase greatly on removal of protein (Figure S1A).

### Anomalous Diffusion Behavior is Modulated by Crowding

**Figure 7:**
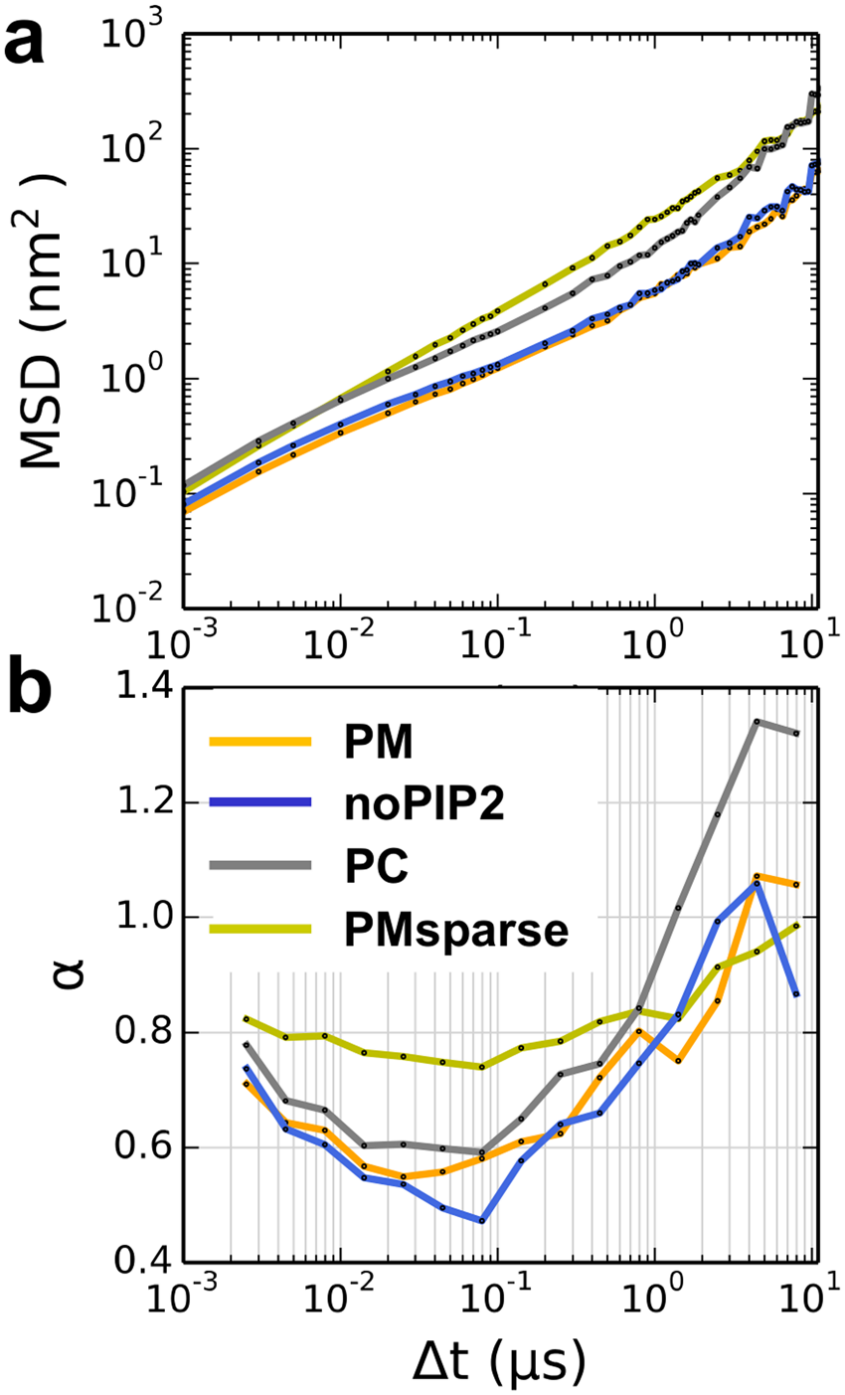
Anomalous diffusion of proteins. (**a**) Log-log plots of mean squared displacement (MSD) as a function of Δt. (**b**) Anomalous exponent α values derived from the gradient of the log-log MSD vs. Δt plots in (**a**).

Protein crowding had a marked effect on the diffusive behavior of the protein (Fig. 7). In all of the crowded simulations (i.e. PM, noPIP2, PC) the value of the anomalous exponent α for proteins initially decreases from 0.7–0.8 at Δt of 1–10 ns to a minimum value of 0.5–0.6 at Δt in the range 10–100 ns. The value of α then increases sharply when measured over timescales of hundreds of nanoseconds, reaching ~1.0 at a timescale of 10 μs. As anticipated, proteins in the more complex lipid mixture simulations (PM and noPIP2) have overall lower anomalous exponents (and are therefore more sub-diffusive) than those of the simple PC membrane. Similar protein behavior is observed in the small simulations (Figure S2). At large timescales (Δt > 1 μs) the protein α values of the PC membrane system start to exceed 1, which may be due to the large protein clusters, in which the proteins tend to move in similar direction, and to a lack of sampling at larger timescales.

When crowding is decreased, as in the PMsparse simulation, the magnitude of the anomalous diffusion coefficient for the protein is higher when measured over timescales <1 μs compared to the crowded PM, and the variation in α as the measurement timescale is varied becomes much less pronounced (the protein α values remain between 0.7 and 0.8 for Δt <1 μs). At larger (Δt) timescales (1–10 μs), sub-diffusive behavior of the PMsparse system becomes more like that of the crowded PM system.

**Figure 8:**
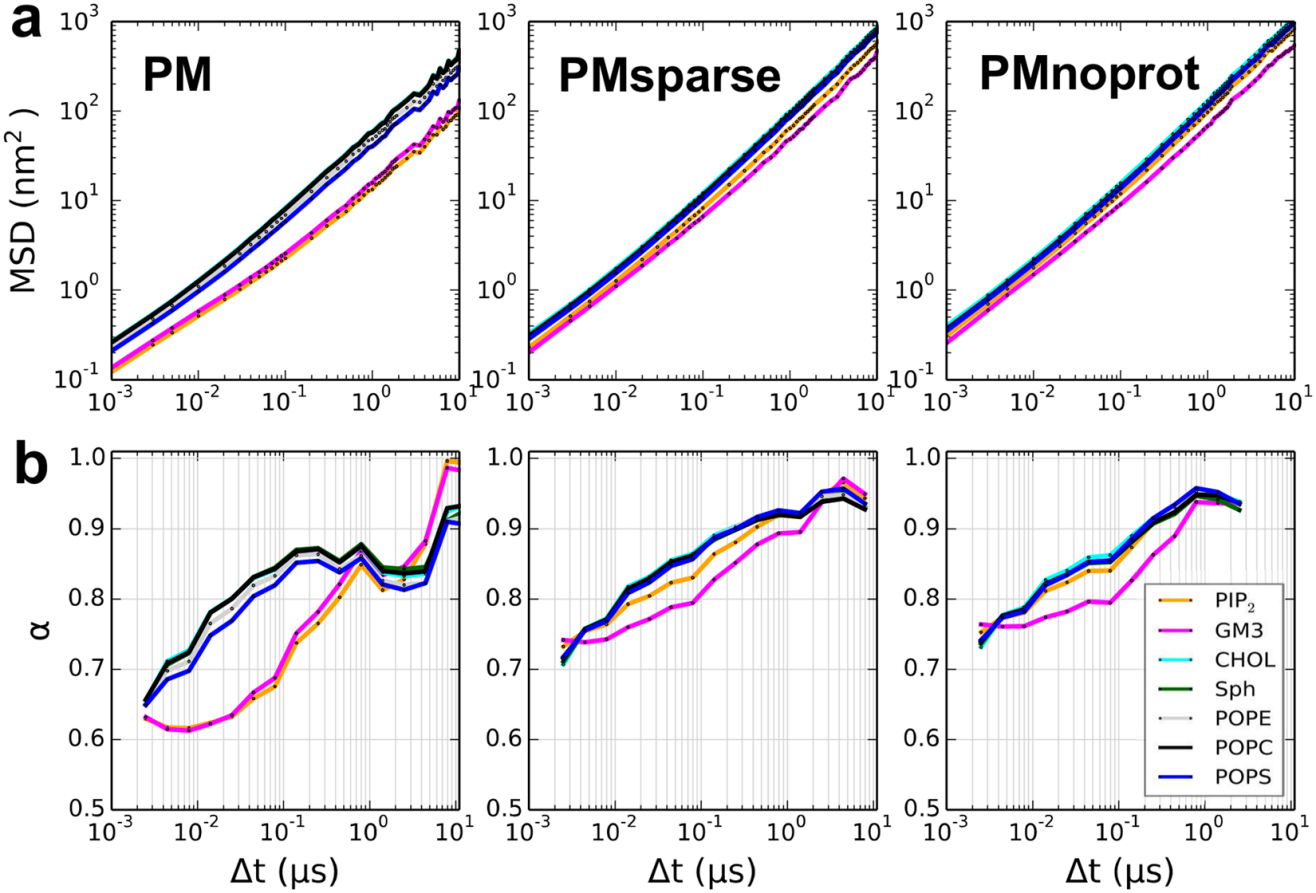
Anomalous diffusion of lipids. (**a**) Log-log plots of mean squared displacement (MSD) as a function of Δt. (**b**) Anomalous exponent α values, calculated by taking the gradient of the log-log MSD vs. Δt plots. Data are shown for all lipid species for the PM, PMsparse and PMnoprot simulations, demonstrating the effect of protein crowding on lipid subdiffusion.

By varying the concentration of the Kir channel in the membrane, the effect of protein-lipid interactions on lipid diffusion can also be inferred (Fig. 8). At high levels of crowding, when almost all the PIP_2_ and GM3 lipids are engaged in protein-lipid interactions, PIP_2_ and GM3 have much lower α values than other lipid species at low Δt timescales (in the order of 10–100 ns), indeed sub-diffusive behavior is similar to that of the protein (Fig. 8 and Figures S1, S2 and S3). However, if protein concentration is decreased (in the PMsparse simulation), the sub-diffusive behavior of PIP_2_ becomes similar to that of other lipid species. In simulations without protein, PIP_2_ α values are indistinguishable from those of other lipid species. GM3, however, continues to have much lower α values than other lipids when proteins are removed. This may be due to GM3 clusters forming in the membrane without the presence of protein^54^, causing marked sub-diffusive behavior of GM3 at the 10–100 ns timescales.

We have observed a complex interplay between the clustering of the Kir channel protein and its diffusive dynamics. In particular, the clustering of proteins in the membrane, driven by crowding, leads to markedly anomalous (and slowed) dynamics of those lipids, which interact most strongly with the channel protein, namely PIP_2_ and GM3. In turn, slower diffusion reduces the rate of protein clustering, resulting in more compact and branched protein clusters.

### Membrane Undulations

**Figure 9:**
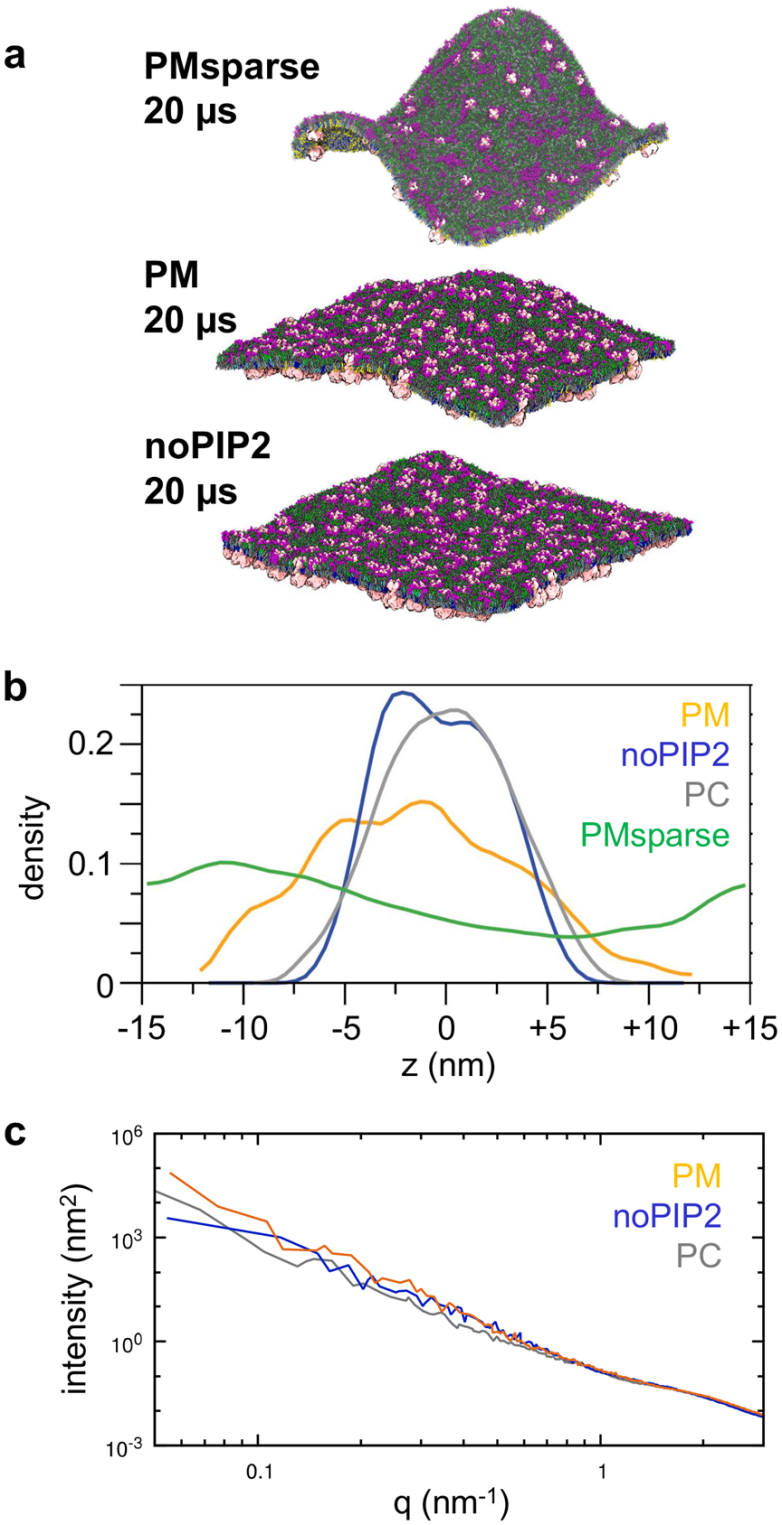
(**a**) Membrane undulations seen in snapshots from the uncrowded PMsparse simulation, and for the PM and noPIP_2_ simulations, viewed from the extracellular side of the respective membranes. Lipids are colored: POPC, dark grey; POPE, light grey; cholesterol, cyan; Sph, green; GM3 (magenta). (**b**) Normalized density of lipid phosphate groups with respect to the z-axis (normal to the membrane at the start of simulations) averaged over 10–20 µs for the PM (yellow-orange), noPIP2 (blue), PC (grey) and PMsparse (green) simulations. (**c**) Power spectra of membrane undulations for the PM (yellow-orange), noPIP2 (blue), and PC (grey) simulations. The spectra were derived from simulation data between 10 and 20 µs.

In previous simulations of a protein-free PM, the membrane was observed to undergo substantial vertical fluctuations leading to undulatory motions, reflecting a significantly reduced bending rigidity of the membrane relative to a simple PC bilayer^23,54^. Such large movements (ca.±15 nm along the bilayer normal) are also seen in the PMsparse simulation (Fig. 9). In contrast, the fluctuations are smaller (ca.±5 nm along the bilayer normal) in the PC and noPIP2 simulations. Interestingly, in the PM membrane (containing 144 Kir molecules) the magnitude of the fluctuations is intermediate between these two extremes. The overall pattern of the magnitude of bilayer undulations is PMsparse >PM >noPIP2 ≈ PC (Fig. 9b).

Simulations of membranes lacking PIP_2_ displayed little change in membrane shape during the course of the simulations (Fig. 9a), with the membrane remaining planar. However, in PM simulations, where compositions were identical except for the presence of PIP_2_, changes in membrane shape were observed, with substantive undulations in the membrane appearing. This propensity for membrane deformation increased as the density of protein, and hence crowding, decreased, indicating that in the PM system a degree of protein crowding and clustering flattened the membrane. The observed effect of PIP_2_ conflicts with studies of simple model membranes formed by e.g. amphiphiles^62^ which indicate that increasing the surface charge on a membrane lead to an increase in bending rigidity. We presume this difference arises because our model membranes do not form a symmetric bilayer and also because their lipids cluster laterally. Thus, both lipid composition (the presence of anionic lipids) and clustering of membrane proteins can modify the bending rigidity of a model membrane.

Membrane undulations were quantified by calculating the power spectra of the height fluctuations of the bilayer (although the asymmetry of the bilayers precludes calculation of the bending modulus from these spectra^23,63^). The power spectra show that for low wavenumbers (q < 0.1 nm^−1^, corresponding to undulations in the range 10–20 nm) there was an effect of lipid composition on the intensity of undulations such that the magnitudes of the undulations followed the order PM >noPIP2 ≈ PC (Fig. 9c). At higher wavenumbers (q > 0.5 nm^−1^, or undulations <2 nm) the power spectra converge, indicating little effect of the different lipid compositions. Thus, the effect of adding of PIP_2_ affects the dynamics of the membrane on a length scale of 5 nm and above, corresponding approximately to the size of a Kir channel molecule.

### Implications

We have revealed a link between the clustering of Kir channel proteins and the anomalous diffusion of both proteins and lipids. Anomalous diffusion has been observed in a number of previous simulation studies of membrane proteins^11,64–66^, and in particular the protein diffusion coefficients observed here agree well with e.g. membrane protein diffusion rates in PC/PG liposomes measured using fluorescence correlation spectroscopy^67^. Our simulations allowed us to analyze the effect of protein crowding and clustering on lipids that interact specifically with the channel protein (PIP_2_ and GM3), and which, in the case of PIP_2_ is an allosteric activator of the channel^68^. Furthermore, our simulations provide an example of a ‘clustered protein lattice’ in a reasonably complex model of a cell membrane, and reveal how such cluster formation may influence both lipid and protein dynamics within a membrane.

Of course, the simulations described remain a simplification of real cell membranes. In particular, the clustering of Kir channels in the simulations is brought about purely by crowding (albeit at ‘physiological’ densities) and other proteins are not present. It is likely that the cytoskeleton immobilizes certain membrane proteins^69–71^, an effect which in the case of Kir channels may be mediated in postsynaptic membranes via PSD-95^38^. Our simulations suggest that such cytoskeletally-mediated clustering may build upon an intrinsic dynamic organization of Kir channels which is brought about by crowding and diffusional restriction in complex cell membranes^72^.

It will be important to understand the impact of channel clustering on Kir channel activity. Given the interactions of (clustered) Kir channels with the activating lipid PIP_2_, it will be of interest to compare our observations in what remains a relatively simple membrane model with behavior in more complex laterally anisotropic cell membranes containing e.g. cholesterol-induced nanodomains. We are also not able to observe large-scale protein conformational changes using CG MD (for instance, those related to channel activation). The gating kinetics of Kir channels, and how this may effect, and be effected by, the crowding of Kir channels is an interesting further question, which will require further work, although a recent communication suggests a link in the case of KcsA^73^. Furthermore, understanding the localization and behavior of complexes of Kir and other channels is directly relevant to a number of disease states, e.g. arrythmogenic cardiac disease^74^.

### Methodological Considerations

A number of technical issues may need to be resolved in order to allow closer comparison to experiments and cellular observations.

The first concerns simulation size effects: diffusion is expected to increase with system size^75,76^, although this is not seen in the simulations presented here. This is perhaps due to there being multiple copies of Kir channels even in the ‘small’ systems and the formation of compartments due to protein clustering in both ‘small’ and ‘large’ systems.

There also may be issues of convergence and of the behaviour of the coarse-grained forcefield in large scale simulations with many interacting proteins^77,78^, and in the forcefield representation of certain lipids, eg. GM3^79^. However, we should note that contrary to some expectations, the MARTINI forcefield seems if anything to *under*estimate the strength of protein/protein interactions within membranes once a rigorous comparison with experimental data is undertaken^80^. We note that in this cited study a different way of accounting for electrostatics is employed from in the current study (PME rather than the switch/cutoff function used here). However, we note that a number of other studies using the switch/cutoff representation of electrostatics have used the MARTINI force field to specifically identify experimentally validated protein-protein interfaces^81–84^. Furthermore, a recent study of the interaction of gangliosides with the WALP transmembrane helix peptide and with aquaporin, showed that, when using polarizable water, PME electrostatics did not have an effect on protein-lipid or lipid-lipid interactions^79^. Whilst the use of polarizable CG water^85^ may address some concerns surrounding CG force fields, the speed-up from atomistic is around three times less than classical MARTINI, thus rendering its use prohibitive in the case of the simulations performed here. It should also be noted that the use of polarizable water, did not improve the protein-lipid interaction in the case of aquaporin and GM3 in a recent study^79^.

In terms of convergence we performed tests based on protein MSDs (see SI Figure S4), which revealed that the protein MSD per frame reaches a plateau once large clusters (involving at least half of all channels in a membrane) formed, suggesting that our simulation lengths were sufficient to probe clustering and the resultant diffusional behavior of both channels and lipids.

## Conclusions

Here we have presented insights into the nanoscale dynamic organization of cell membranes obtained from large-scale coarse-grained simulations of multiple Kir channels in model membranes at physiological degrees of protein crowding. Clustering of the channel proteins was modulated by the compositional complexity of the lipid bilayer, which altered not only the dynamics but also the spatiotemporal patterns of cluster formation.

Diffusion coefficients for both protein and lipid were lowest in the most complex lipid mixtures. Removing cholesterol caused the largest increase in diffusion coefficients, as might be anticipated^57,58^. Anomalous diffusion was observed for all proteins and lipids, and the anomalous diffusion of those lipids, which interacted strongly with the protein, was most affected by channel crowding; protein clustering slowed and perturbed diffusion of the interacting lipids (PIP_2_ and GM3). Protein crowding and clustering also altered the mechanical properties of the bilayer (for instance, bilayer fluctuations) in a PIP_2_ dependent fashion.

These simulations provide us with a glimpse of the complexities inherent in the interplay between the dynamic organization of Kir channels and of their allosteric activator lipid PIP_2_^86^ in cell membranes. As we have demonstrated, protein clustering can slow and cluster lipid modulators and form dynamic compartments in the membrane, which in turn may play an important role in the kinetics of channel activation^68^. More generally, our results imply that in order to understand the biological function of a cell membrane one must fully characterize the dynamic organization of the proteins and lipids on a nano to microscale.

## Methods

### System set-up and simulations performed

Simulations performed are shown in Table 1 and Table S1. The Kir2.2 channel atomistic structure (PDB ID: 3SPI^32^); was converted to a coarse-grained representation using the martinize.py script (version 2.4) downloaded from the MARTINI website (http://md.chem.rug.nl/index.php/tools2/proteins-and-bilayers) with the MARTINI2.2 force field^87,88^ and the ElNeDyn elastic network model^89^. A single CG model of Kir2.2 was embedded into a phosphatidyl choline (PC) bilayer by allowing lipids to self-assemble around the protein in a short simulation^90^. The PC membrane was subsequently converted into a membrane of the desired lipid composition using an in-house exchange lipid methodology^54^. The Kir2.2 channel in a model plasma membrane (PM) was then equilibrated for 100 ns prior to being tessellated, using the GROMACS tool genconf, to form 12 × 12, 6 × 6 and 3 × 3 grids. The resulting systems contained either 144, 36 or 9 Kir2.2 channels, ~55,000 (12 × 12 and 6 × 6 systems) or ~3,500 (3 × 3 systems) lipids, and were solvated using the standard MARTINI water model and neutralized to a 0.15 M NaCl concentration (see Table 1 for details).

Lipid bilayers were modeled with a mixture of the most abundant lipids present in mammalian cell plasma membrane^2–4^, with lipids distributed asymmetrically between the inner and outer leaflets, as previously described^54,55^. In the PM model, which is the most complex presented here, the outer leaflet contained phosphatidyl choline (PC)/phosphatidyl ethanolamine (PE)/sphingolipid (Sph)/ganglioside (GM3)/cholesterol (Chol) lipids in a ratio of 40:10:15:10:25, while the inner leaflet contained PC/PE/phosphatidyl serine (PS)/phosphatidylinositol 4,5-bisphosphate (PIP_2_)/Chol in a ratio of 10:40:15:10:25. PC, PE, and PS were modeled with 1-palmitoyl-2-oleoyl (i.e., PO) lipid tails. PIP_2_ was removed from the PM model by substitution with PE, as these two species clustered in areas of higher membrane curvature in previous PM simulations^54^. In simulations where lipid complexity was further reduced, the proportions of the PM simulation were maintained for all remaining lipid species (Table 1 and Table S1). Sph and GM3 were modeled with a monounsaturated ceramide tail; PIP_2_ was modeled using fully saturated tails.

### Simulation parameters

All simulations were performed using GROMACS 4.6 (www.gromacs.org) and the standard MARTINI protocol. Periodic boundary conditions were applied, and a time step of 20 fs was used in all simulations. The temperature was maintained at 323 K using a Berendsen thermostat^91^, and the pressure at 1 bar using a Berendsen barostat, except in the case of the PCnoprot simulation, where a velocity rescale thermostat^92^ and Parinello-Rahman barostat^93^ were used. For both the temperature and pressure, a coupling constant of 4 ps was used for all simulations containing protein and 1 ps for the PMnoprot and PCnoprot simulations. In all simulations, the reaction field coulomb type was used with a switching function from 0.0 to 1.2 nm, and the van der Waals interactions were cutoff at 1.2 nm with a switching function applied from 0.9 nm. The LINCS algorithm^94^ was used to constrain covalent bonds to their equilibrium values. All systems containing protein were simulated for between 20 and 50 μs, whereas systems without proteins were performed for 10 μs (PMnoprot) or 5 μs (PCnoprot); see Table 1.

### Simulation analysis

Radial distribution functions of lipid headgroups around the channels were calculated using the GROMACS tool, g_rdf. Clustering analysis was performed using in-house Python scripts, making use of the NumPy^95^, MDAnalysis^96,97^ and NetworkX^98^ Python libraries. In the clustering algorithm, proteins were considered to be interacting when the centroids of the cytoplasmic domains (defined as residues 1–45 and 141–377) were within 8.2 nm of one another. Protein-protein interactions were identified using in-house clustering scripts, again making use of the NumPy and MDAnalysis modules. Residues of neighboring proteins were considered to be interacting when residue centroids were within 0.7 nm of one another. Phosphate densities were calculated using the g_density tool from GROMACS.

Diffusion analysis was carried out on trajectories with center of mass motion of the protein and lipids removed, and with the first 50 ns removed. Although the PM simulation was run for 50 µs, only the first 20 µs was used for diffusion analysis, in order to be comparable with analysis of the NoPIP2, PC, PMsparse and small simulations. Time- and ensemble-average mean squared displacement (MSD) for each species was extracted using a time step of 1 ns. Diffusion coefficients (D_α_) were obtained by fitting the equation MSD = 4D_α_ Δt^α^ to graphs of MSD vs Δt, with Δt in the range 1 ns to 2 µs. Error bars show the standard deviation for the nonlinear fitting procedure. Scripts to perform this analysis were from https://github.com/tylerjereddy/diffusion_analysis_MD_simulations ^99^. Alpha values were obtained by fitting a straight line at fixed intervals to the log-log plots of MSD vs Δt.

Power spectra of membrane undulations were estimated as detailed in^23^. The surface of the bilayer was calculated using lipid phosphate beads projected onto a grid of size 0.5 nm. The Fourier transform of the bilayer surface was calculated using FFTW routines in the SciPy Python library^100^, and 1D power spectra obtained by performing radial averaging. The code can be obtained from GitHub (https://github.com/philipwfowler/calculate-bilayer-power-spectrum).

Graphs were plotted using gnuplot 4.6 (www.gnuplot.info) and Matplotlib^101^ and molecular visualization used VMD^102^.

## Electronic supplementary material

Supplementary Information

## References

[1] Dupuy AD, Engelman DM (2008). Protein area occupancy at the center of the red blood cell membrane. Proc. Natl. Acad. Sci. USA.

[2] van Meer G, Voelker DR, Feigenson GW (2008). Membrane lipids: where they are and how they behave. Nature Rev. Mol. Cell Biol..

[3] van Meer G, de Kroon AIPM (2011). Lipid map of the mammalian cell. J. Cell Sci..

[4] Coskun, Ü. & Simons, K. Cell membranes: the lipid perspective. *Structure* 1543–1548 (2011). 10.1016/j.str.2011.10.010 22078554

[5] Linden, M., Sens, P. & Phillips, R. Entropic tension in crowded membranes. *PLoS Comp. Biol*. **8** (2012). 10.1371/journal.pcbi.1002431 PMC3305330 22438801

[6] Rassam P (2015). Supramolecular assemblies underpin turnover of outer membrane proteins in bacteria. Nature.

[7] Guigas G, Weiss M (2016). Effects of protein crowding on membrane systems. Biochim. Biophys. Acta Biomembranes.

[8] Garcia-Parajo MF, Cambi A, Torreno-Pina JA, Thompson N, Jacobson K (2014). Nanoclustering as a dominant feature of plasma membrane organization. J. Cell Sci..

[9] Saka, S. K. *et al*. Multi-protein assemblies underlie the mesoscale organization of the plasma membrane. *Nature Comms*. **5** (2014). 10.1038/ncomms5509 PMC4124874 25060237

[10] Niemela PS (2010). Membrane proteins diffuse as dynamic complexes with lipids. J. Amer. Chem. Soc..

[11] Javanainen M (2013). Anomalous and normal diffusion of proteins and lipids in crowded lipid membranes. Faraday Disc..

[12] Goose JE, Sansom MSP (2013). Reduced lateral mobility of lipids and proteins in crowded membranes. PLoS Comp. Biol..

[13] Houser JR (2016). The impact of physiological crowding on the diffusivity of membrane bound proteins. Soft Matter.

[14] Lee AG (2011). Biological membranes: the importance of molecular detail. Trends Biochem. Sci..

[15] Laganowsky A (2014). Membrane proteins bind lipids selectively to modulate their structure and function. Nature.

[16] Yeagle PL (2014). Non-covalent binding of membrane lipids to membrane proteins. Biochim. Biophys. Acta.

[17] Eggeling C, Honigmann A (2016). Closing the gap: the approach of optical and computational microscopy to uncover biomembrane organization. Biochim. Biophys. Acta Biomembranes.

[18] Hedger G, Sansom MSP (2016). Lipid interaction sites on channels, transporters and receptors: recent insights from molecular dynamics simulations. Biochim. Biophys. Acta.

[19] Manna M (2016). Mechanism of allosteric regulation of β2-adrenergic receptor by cholesterol. eLife.

[20] Lingwood D, Simons K (2010). Lipid rafts as a membrane-organizing principle. Science.

[21] Fowler PW, Williamson JJ, Sansom MSP, Olmsted PD (2016). Roles of inter-leaflet coupling and hydrophobic mismatch in lipid membrane phase-separation kinetics. J. Amer. Chem. Soc..

[22] Vanni S, Hirose H, Barelli H, Antonny B, Gautier R (2014). A sub-nanometre view of how membrane curvature and composition modulate lipid packing and protein recruitment. Nature Comms..

[23] Fowler PF (2016). Membrane stiffness is modified by integral membrane proteins. Soft Matter.

[24] Bernardino de la Serna J, Schütz GJ, Eggeling C, Cebecauer M (2016). There is no simple model of the plasma membrane organization. Front. Cell. Dev. Biol..

[25] Marrink SJ, Tieleman DP (2013). Perspective on the Martini model. Chem. Soc. Rev..

[26] Ingolfsson HI, Arnarez C, Periole X, Marrink SJ (2016). Computational ‘microscopy’ of cellular membranes. J. Cell. Sci..

[27] Chavent M, Duncan AL, Sansom MSP (2016). Molecular dynamics simulations of membrane proteins and their interactions: from nanoscale to mesoscale. Curr. Opin. Struct. Biol..

[28] D’Avanzo N, Lee S-J, Cheng WWL, Nichols CG (2013). Energetics and location of phosphoinositide binding in human Kir2.1 channels. J. Biol. Chem..

[29] Lee, S.-J. *et al*. Secondary anionic phospholipid binding site and gating mechanism in Kir2.1 inward rectifier channels. *Nature Comms*. **4** (2013). 10.1038/ncomms3786 PMC3868208 24270915

[30] Fürst O, Nichols CG, Lamoureux G, D’Avanzo N (2014). Identification of a cholesterol-binding pocket in inward rectifier K^+^ (Kir) channels. Biophys. J..

[31] Hibino H (2010). Inwardly rectifying potassium channels: their structure, function, and physiological roles. Physiol. Rev..

[32] Hansen SB, Tao X, Mackinnon R (2011). Structural basis of PIP_2_ activation of the classical inward rectifier K^+^ channel Kir2.2. Nature.

[33] Schmidt MR, Stansfeld PJ, Tucker SJ, Sansom MSP (2013). Simulation-based prediction of phosphatidylinositol 4,5-bisphosphate binding to an ion channel. Biochem..

[34] Cheng WWL, D’Avanzo N, Doyle DA, Nichols CG (2011). Dual-mode phospholipid regulation of human inward rectifying potassium channels. Biophys. J..

[35] Lee SJ (2016). Structural basis of control of inward rectifier Kir2 channel gating by bulk anionic phospholipids. J. Gen. Physiol..

[36] D’Avanzo N, Hyrc K, Enkvetchakul D, Covey DF, Nichols CG (2011). Enantioselective protein-sterol interactions mediate regulation of both prokaryotic and eukaryotic inward rectifier K^+^ channels by cholesterol. PLoS ONE.

[37] Rosenhouse-Dantsker, A., Noskov, S., Durdagi, S., Logothetis, D. E. & Levitan, I. Identification of novel cholesterol-binding regions in Kir2 channels. *J. Biol. Chem*. (2013). 10.1074/jbc.M113.496117 PMC3829427 24019518

[38] Fomina S (2011). Self-directed assembly and clustering of the cytoplasmic domains of inwardly rectifying Kir2.1 potassium channels on association with PSD-95. Biochim. Biophys. Acta Biomembranes.

[39] Freeman SA, Desmazieres A, Fricker D, Lubetzki C, Sol-Foulon N (2016). Mechanisms of sodium channel clustering and its influence on axonal impulse conduction. Cell. Molec. Life Sci..

[40] Molina ML (2015). Competing lipid-protein and protein-protein interactions determine clustering and gating patterns in the potassium channel from *Streptomyces lividans* (KcsA). J. Biol. Chem..

[41] Raja M, Vales E (2010). Mutations in the K^+^-channel KcsA toward Kir channels alter salt-induced clusterization and blockade by quaternary alkylammonium ions. J. Membr. Biol..

[42] Molina ML (2006). Clustering and coupled gating modulate the activity in KcsA, a potassium channel model. J. Biol. Chem..

[43] Grage SL (2011). Bilayer-mediated clustering and functional interaction of mscl channels. Biophy. J..

[44] Taverna E (2004). Role of lipid microdomains in P/Q-type calcium channel (Ca(v)2.1) clustering and function in presynaptic membranes. J. Biol. Chem..

[45] Wang SSH, Thompson S (1992). A-type potassium channel clusters revealed using a new statistical-analysis of loose patch data. Biophys. J..

[46] Ianoul A (2004). Near-field scanning fluorescence microscopy study of ion channel clusters in cardiac myocyte membranes. Biophys. J..

[47] Kim E, Niethammer M, Rothschild A, Jan YN, Sheng M (1995). Clustering of Shaker-type K^+^ channels by interaction with a family of membrane-associated guanylate kinases. Nature.

[48] Wong W, Newell EW, Jugloff DGM, Jones OT, Schlichter LC (2002). Cell surface targeting and clustering interactions between heterologously expressed PSD-95 and the Shal voltage-gated potassium channel, Kv4.2. J. Biol. Chem..

[49] Cohen NA, Brenman JE, Snyder SH, Bredt DS (1996). Binding of the inward rectifier K+ channel Kir 2.3 to PSD-95 is regulated by protein kinase A phosphorylation. Neuron.

[50] Horio Y (1997). Clustering and enhanced activity of an inwardly rectifying potassium channel, Kir4.1, by an anchoring protein, PSD-95/SAP90. J. Biol. Chem..

[51] Nehring RB (2000). Neuronal inwardly rectifying K+ channels differentially couple to PDZ proteins of the PSD-95/SAP90 family. J. Neurosci..

[52] Inanobe A (2002). Inward rectifier K+ channel Kir2.3 is localized at the postsynaptic membrane of excitatory synapses. Amer. J. Physiol. Cell Physiol..

[53] Pegan S (2007). NMR studies of interactions between C-terminal tail of Kir2.1 channel and PDZ1,2 domains of PSD95. Biochem..

[54] Koldsø H, Shorthouse D, Hélie J, Sansom MSP (2014). Lipid clustering correlates with membrane curvature as revealed by molecular simulations of complex lipid bilayers. *PLoS Comp*. Biol..

[55] Koldsø H, Sansom MSP (2015). Organization and dynamics of receptor proteins in a plasma membrane. J. Amer. Chem. Soc..

[56] Hedger G, Koldsø H, Sansom MSP (2016). Free energy landscape of lipid interactions with regulatory binding sites on the transmembrane domain of the EGF receptor. J. Phys. Chem. B.

[57] Filippov A, Orädd G, Lindblom G (2003). The effect of cholesterol on the lateral diffusion of phospholipids in oriented bilayers. Biophys. J..

[58] Jeon, J. H., Monne, H. M. S., Javanainen, M. & Metzler, R. Anomalous diffusion of phospholipids and cholesterols in a lipid bilayer and its origins. *Phys. Rev. Lett*. **109** (2012). 10.1103/PhysRevLett.109.188103 23215336

[59] Grouleff J, Irudayam SJ, Skeby KK, Schiott B (2015). The influence of cholesterol on membrane protein structure, function, and dynamics studied by molecular dynamics simulations. Biochimica Et Biophysica Acta-Biomembranes.

[60] Slotte JP (2013). Biological functions of sphingomyelins. Progress Lipid Res..

[61] Kraft ML (2017). Sphingolipid organization in the plasma membrane and the mechanisms that influence it. Front Cell. Dev. Biol..

[62] Bradbury R, Nagao M (2016). Effect of charge on the mechanical properties of surfactant bilayers. Soft Matter.

[63] Brown FLH (2008). Elastic Modeling of biomembranes and lipid bilayers. Ann. Rev. Phys. Chem..

[64] Poyry S (2013). Atomistic simulations indicate cardiolipin to have an integral role in the structure of the cytochrome bc(1) complex. Biochim. Biophys. Acta.

[65] Jeon JH, Javanainen M, Martinez-Seara H, Metzler R, Vattulainen I (2016). Protein crowding in lipid bilayers gives rise to non-Gaussian anomalous lateral diffusion of phospholipids and proteins. Phys. Rev. X.

[66] Metzler R, Jeon JH, Cherstvy AG (2016). Non-Brownian diffusion in lipid membranes: Experiments and simulations. Biochim. Biophys. Acta Biomembranes.

[67] Ramadurai S (2009). Lateral diffusion of membrane proteins. J. Amer. Chem. Soc..

[68] Hansen SB (2015). Lipid agonism: The PIP2 paradigm of ligand-gated ion channels. Biochim. Biophys. Acta.

[69] Kusumi A, Suzuki KGN, Kasai RS, Ritchie K, Fujiwara TK (2011). Hierarchical mesoscale domain organization of the plasma membrane. Trends Biochem. Sci..

[70] Heinemann F, Vogel SK, Schwille P (2013). Lateral membrane diffusion modulated by a minimal actin cortex. Biophys. J..

[71] Goiko M, de Bruyn JR, Heit B (2016). Short-lived cages restrict protein diffusion in the plasma membrane. Sci. Reports.

[72] Koldsø H, Reddy T, Fowler PW, Duncan AL, Sansom MSP (2016). Membrane compartmentalization reducing the mobility of lipids and proteins within a model plasma membrane. J. Phys. Chem. B..

[73] Visscher, K. M. *et al*. Supramolecular organization and functional implications of K^+^ channel clusters in membranes. *Angewandte Chemie Int. Edn*. **56**, 13222–13227 (2017). 10.1002/anie.201705723 PMC5655921 28685953

[74] Willis BC, Ponce-Balbuena D, Jalife J (2015). Protein assemblies of sodium and inward rectifier potassium channels control cardiac excitability and arrhythmogenesis. Amer. J. Physiol. Heart Circul. Physiol..

[75] Camley BA, Lerner MG, Pastor RW, Brown FLH (2015). Strong influence of periodic boundary conditions on lateral diffusion in lipid bilayer membranes. J. Chem. Phys..

[76] Vogele M, Hummer G (2016). Divergent Diffusion Coefficients in Simulations of Fluids and Lipid Membranes. J. Phys. Chem. B.

[77] van Eerden FJ, de Jong DH, de Vries AH, Wassenaar TA, Marrink SJ (2015). Characterization of thylakoid lipid membranes from cyanobacteria and higher plants by molecular dynamics simulations. Biochim. Biophys. Acta.

[78] Wassenaar TA, Ingolfsson HI, Boeckmann RA, Tieleman DP, Marrink SJ (2015). Computational lipidomics with Insane: a versatile tool for generating custom membranes for molecular simulations. J. Chem. Theor. Comput..

[79] Gu RX, Ingolfsson HI, de Vries AH, Marrink SJ, Tieleman DP (2017). Ganglioside-lipid and ganglioside-protein interactions revealed by coarse-grained and atomistic molecular dynamics simulations. J. Phys. Chem. B.

[80] Domański J, Hedger G, Best R, Stansfeld PJ, Sansom MSP (2017). Convergence and sampling in determining free energy landscapes for membrane protein association. J. Phys. Chem. B..

[81] Sengupta D, Marrink SJ (2010). Lipid-mediated interactions tune the association of glycophorin A helix and its disruptive mutants in membranes. Phys. Chem. Chem. Phys..

[82] Periole X, Knepp AM, Sakmar TP, Marrink SJ, Huber T (2012). Structural determinants of the supramolecular organization of G protein-coupled receptors in bilayers. J. Amer. Chem. Soc..

[83] Castillo N, Monticelli L, Barnoud J, Tieleman DP (2013). Free energy of WALP23 dimer association in DMPC, DPPC, and DOPC bilayers. Chem. Phys. Lipids.

[84] Lelimousin M, Limongelli V, Sansom MSP (2016). Conformational changes in the epidermal growth factor receptor: role of the transmembrane domain investigated by coarse-grained metadynamics free energy landscape calculations. J. Amer. Chem. Soc..

[85] Yesylevskyy SO, Schäfer LV, Sengupta D, Marrink SJ (2010). Polarizable water model for the coarse-grained MARTINI force field. *PLoS Comp*. Biol..

[86] Suh BC, Hille B (2008). PIP_2_ is a necessary cofactor for ion channel function: How and why?. Ann. Rev. Biophys..

[87] Monticelli L (2008). The MARTINI coarse grained force field: extension to proteins. J. Chem. Theor. Comp..

[88] de Jong DH (2013). Improved parameters for the Martini coarse-grained protein force field. J. Chem. Theor. Comput..

[89] Periole X, Cavalli M, Marrink SJ, Ceruso MA (2009). Combining an elastic network with a coarse-grained molecular force field: structure, dynamics, and intermolecular recognition. J. Chem. Theory Comput..

[90] Stansfeld PJ (2015). MemProtMD: automated insertion of membrane protein structures into explicit lipid membranes. Structure.

[91] Berendsen HJC, Postma JPM, van Gunsteren WF, DiNola A, Haak JR (1984). Molecular dynamics with coupling to an external bath. J. Chem. Phys..

[92] Bussi G, Donadio D, Parrinello M (2007). Canonical sampling through velocity rescaling. J. Chem. Phys..

[93] Parrinello M, Rahman A (1981). Polymorphic transitions in single-crystals - a new molecular-dynamics method. J. Appl. Phys..

[94] Hess B, Bekker H, Berendsen HJC, Fraaije JGEM (1997). LINCS: A linear constraint solver for molecular simulations. J. Comp. Chem..

[95] van der Walt S, Colbert SC, Varoquaux G (2011). The NumPy Array: A Structure for Efficient Numerical Computation. Computing in Science & Engineering.

[96] Michaud-Agrawal N, Denning EJ, Woolf TB, Beckstein O (2011). MDAnalysis: a toolkit for the analysis of molecular dynamics simulations. J. Comput. Chem..

[97] Gowers, R. J. *et al*. In *Proceedings of the 15th Python in Science conference (SciPy 2016)*. (eds S. Benthall & S. Rostrup) 102–109.

[98] Hagberg, A., Schult, D. & Swart, P. In *Proceedings of the 7th Python in Science conference (SciPy 2008)*. (eds G. Varoquaux, T. Vaught, & J. Millman) 11–15.

[99] Reddy T (2015). Nothing to sneeze at: a dynamic and integrative computational model of an influenza A virion. Structure.

[100] Oliphant TE (2007). Python for scientific computing. Computing in Science & Engineering.

[101] Hunter JD (2007). Matplotlib: A 2D graphics environment. Computing in Science & Engineering.

[102] Humphrey W, Dalke A, Schulten K (1996). VMD - Visual MolecularDynamics. J. Molec. Graph..

