# Supplementary material for: Protein crowding and lipid complexity influence the nanoscale dynamic organization of ion channels in cell membranes
Source: Sci Rep. 2017 Nov 30;7:16647. doi: 10.1038/s41598-017-16865-6 (PMC5709381; doi:10.1038/s41598-017-16865-6)
Supplement: Supplementary file 1 — Supplementary Information [file 41598_2017_16865_MOESM1_ESM.pdf]

Supplementary Information for:

**Protein crowding and lipid complexity influence the nanoscale dynamic organization of ion channels in cell membranes**

*Anna L. Duncan, Tyler Reddy, Heidi Koldsø, Jean Hélie, Philip W. Fowler, Matthieu Chavent & Mark S.P. Sansom*

**Supplementary Figures**

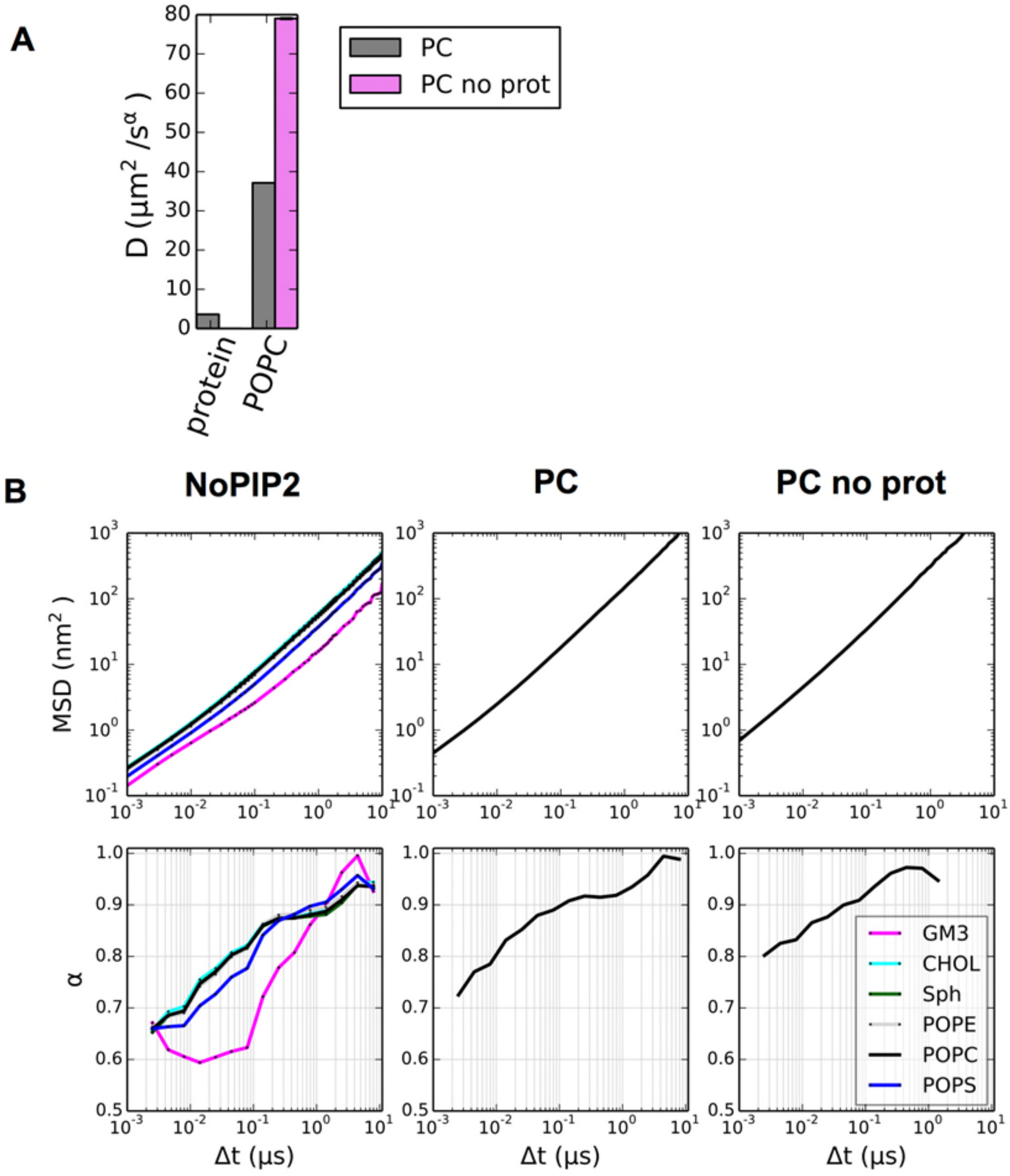


*Figure S1.*

**A** Diffusion coefficient of the Kir protein and POPC in the PC (grey) and the PCnoprot (pink) simulations. Diffusion values are also listed in Table S2. **B** Log-log plots of lipid mean squared displacement (MSD) as a function of Δt (top row) and anomalous exponent α values (bottom row) for the noPIP$_2$, PC and PCnoprot simulations.

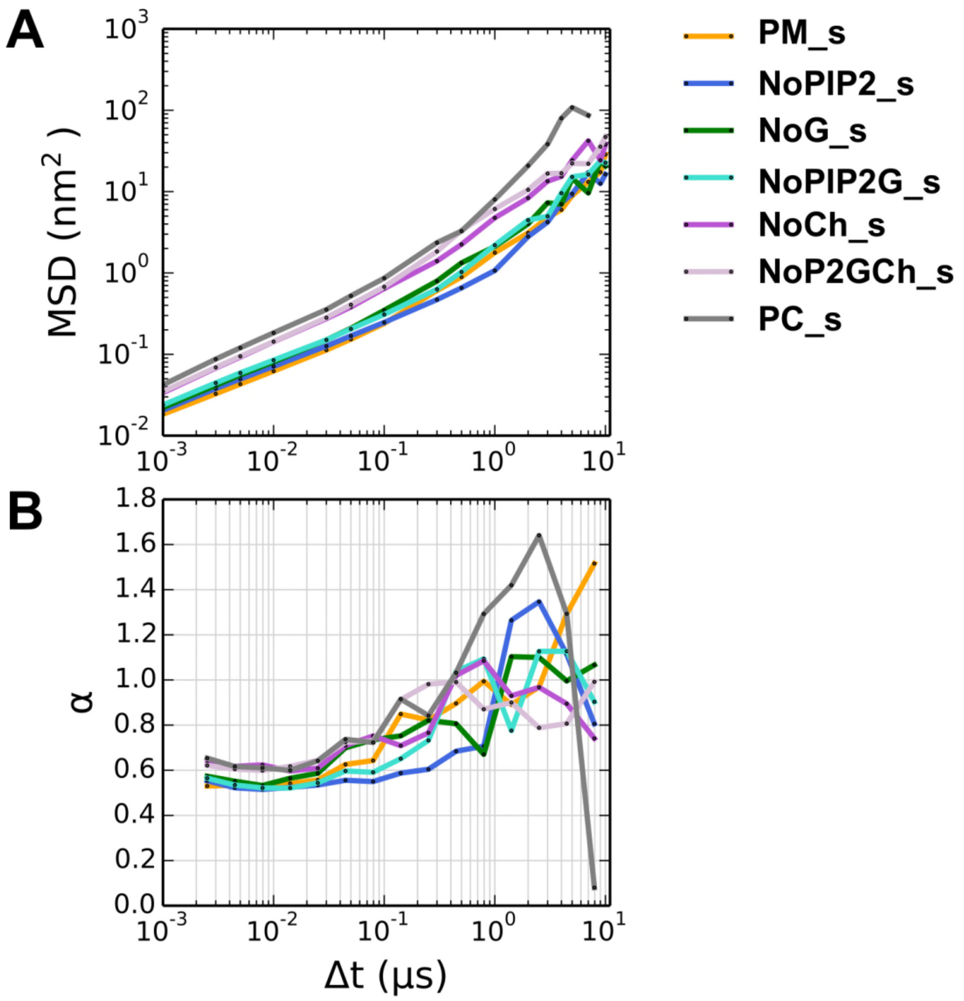


*Figure S2.*

Anomalous diffusion of proteins in ‘small’ simulations. **A** Log-log plots of mean squared displacement (MSD) as a function of Δt . **B** Anomalous exponent α values derived from the gradient of the log-log MSD vs. Δt plots in **A**.

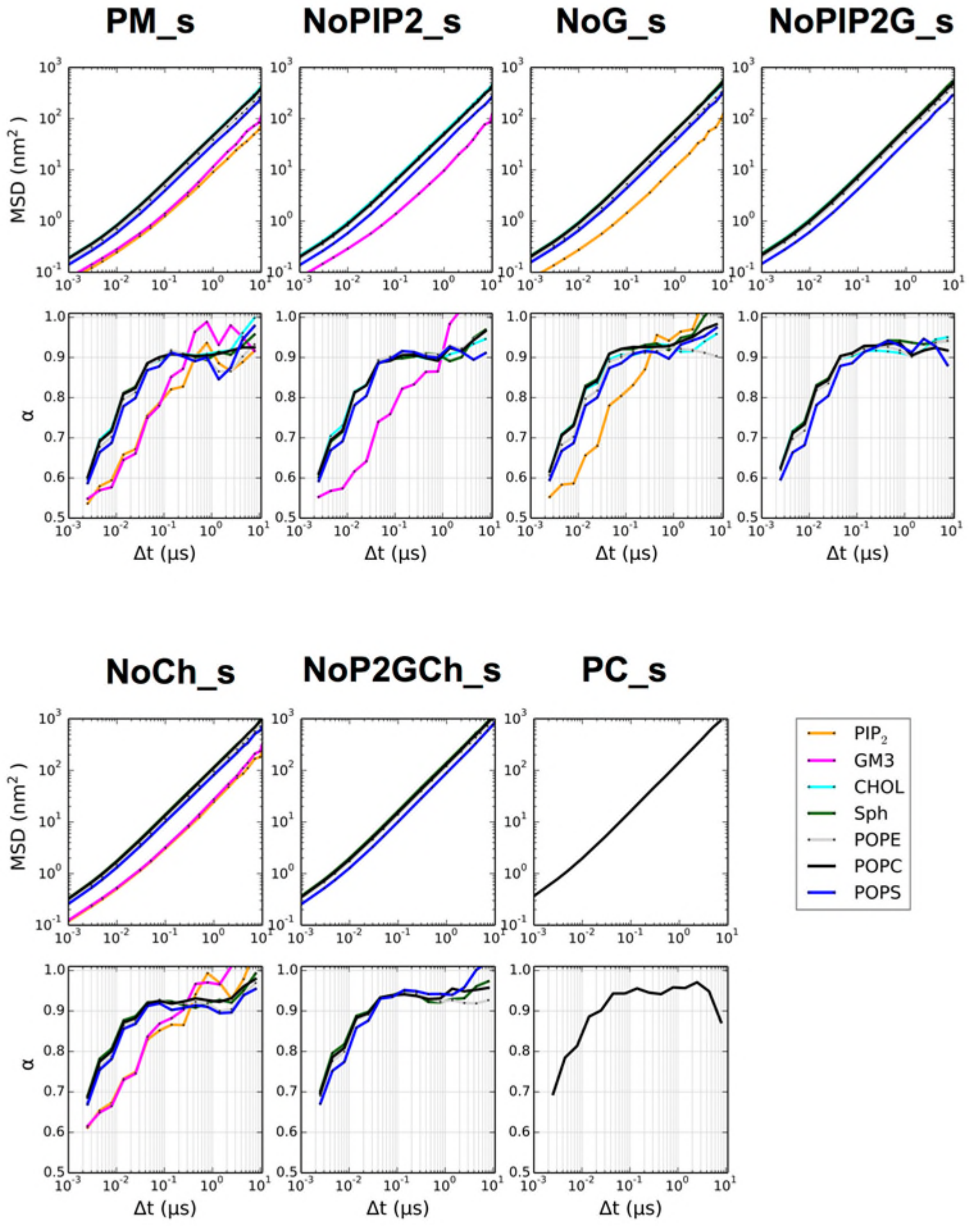


*Figure S3.*

Anomalous diffusion of lipids in 'small' simulations. For each simulation, shown are: log-log plots of mean squared displacement (MSD) as a function of Δt (top); and anomalous exponent α values, calculated by taking the gradient of the log-log MSD vs. Δt plots (bottom). Data are shown for all lipid species for the PM_s, noPIP2_s, noG_s, noPIP2G_s, noCh_s, noP2GCh_s, and PC_s simulations, demonstrating the effect of lipid content on lipid subdiffusion. Lipid species are coloured as shown in the key.

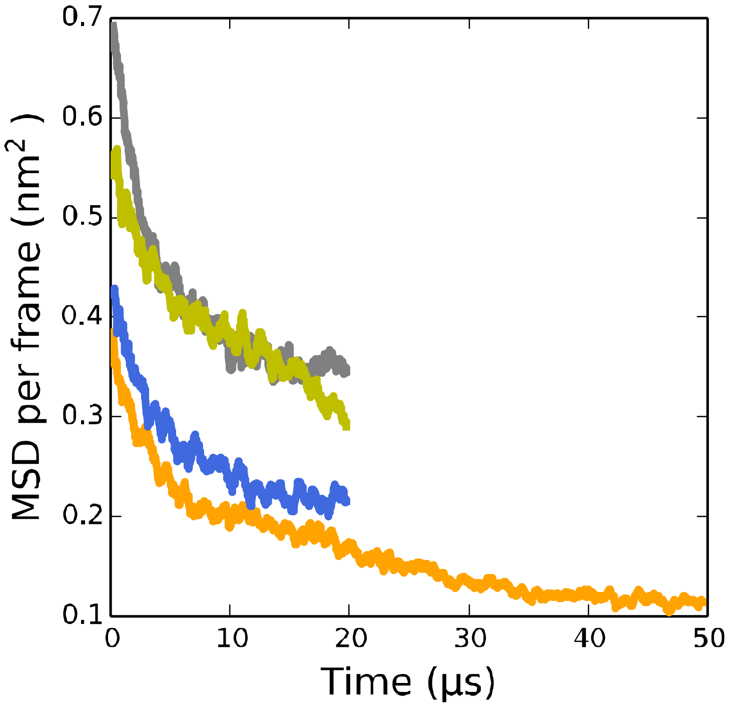


*Figure S4.*

Convergence of large simulations, shown by protein mean squared displacement (MSD) per 5 ns frame as a function of simulation time for PM (orange-yellow), noPIP$_2$ (blue), PC (grey) and uncrowded PMsparse (lime green) simulations.

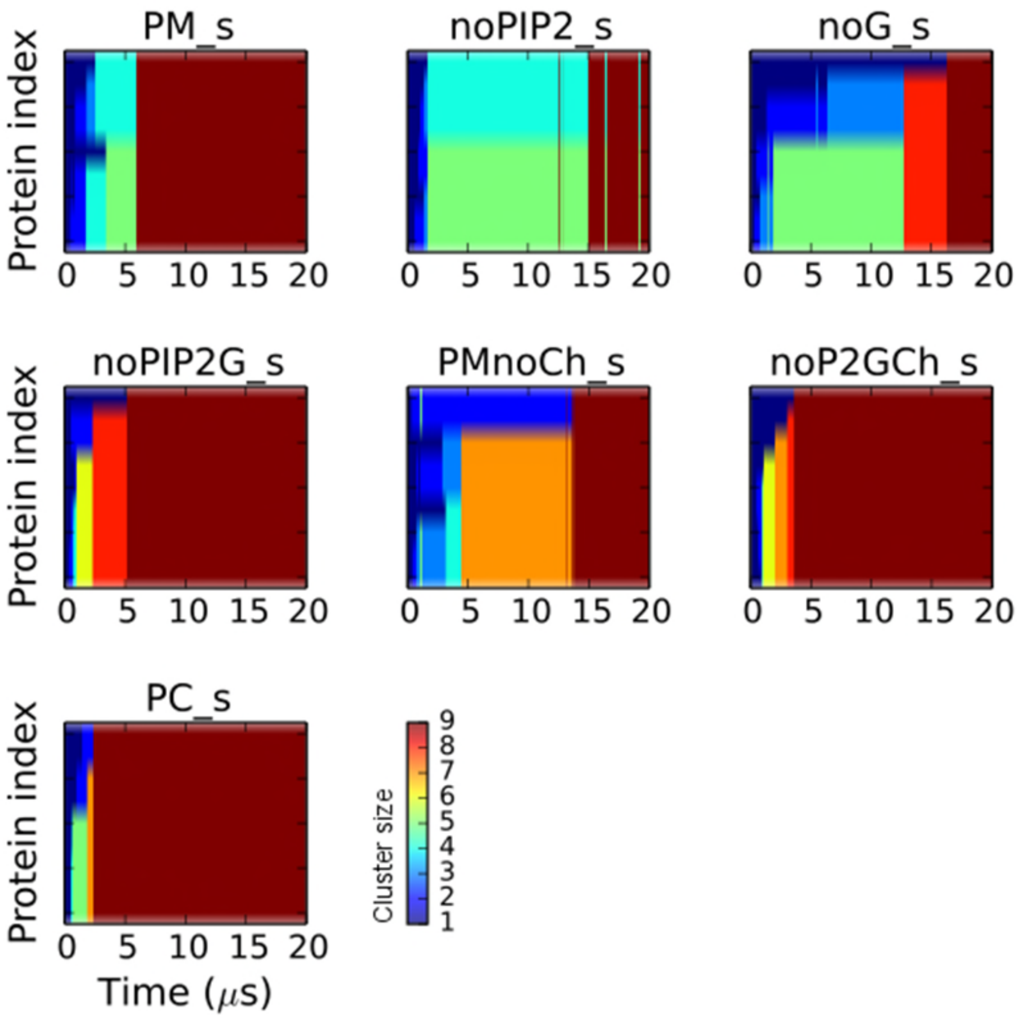


*Figure S5.*

Evolution of Kir clusters over simulation time for the small simulations. A color code is used to indicate the cluster size ranging from deep blue (single channels) to deep red (clusters of 9 channels). The systems are labelled as specified in Table 1.

*Table S1: Bilayer compositions*

| Simulation | Number of proteins | Bilayer composition* |
|---|---|---|
| *Large Systems (~55,000 lipids; ~3.5 M particles)* | | |
| PM | 144 | Inner leaflet: PC:PE:PS:PIP$_2$ :CHOL (10:40:15:10:25)<br>Outer leaflet: PC:PE:Sph:GM3:CHOL (40:10:15:10:25) |
| noPIP2 | 144 | Inner leaflet: PC:PE:PS:CHOL (10:50:15:25)<br>Outer leaflet: PC:PE:Sph:GM3:CHOL (40:10:15:10:25) |
| PC | 144 | PC |
| PMsparse | 36 | As PM |
| PMnoprot | 0 | As PM |
| PCnoprot | 0 | PC |
| *Small Systems (~3500 lipids; ~0.2 M particles)* | | |
| PM_s | 9 | As PM |
| noPIP2_s | 9 | As noPIP2 |
| noG_s | 9 | Inner leaflet: PC:PE:PS:PIP$_2$ :CHOL (10:50:15:10:25)<br>Outer leaflet: PC:PE:Sph:CHOL (50:10:15:25) |
| noPIP2G_s | 9 | Inner leaflet: PC:PE:PS:CHOL (10:50:15:25)<br>Outer leaflet: PC:PE:Sph:CHOL (50:10:15:25) |
| noCh_s | 9 | Inner leaflet: PC:PE:PS:PIP$_2$ :CHOL (10:40:15:10)<br>Outer leaflet: PC:PE:Sph:GM3:CHOL (40:10:15:10) |
| noP2GCh_s | 9 | Inner leaflet: PC:PE:PS:CHOL (10:50:15)<br>Outer leaflet: PC:PE:Sph:CHOL (50:10:15) |
| PC_s | 9 | PC |

*Table S2: Diffusion coefficients*

Diffusion coefficients, as plotted in Figures 5A and B, Figure 6C and Figure S1A.

| **Simulation** | **Diffusion coefficient ($\mu m^2/s^\alpha$)** | | | | | | | |
|---|---|---|---|---|---|---|---|---|
| | Protein | $PIP_2$ | GM3 | POPS | POPE | Sph | Chol | POPC |
| *Large Systems* | | | | | | | | |
| PM | 1.48 ± 0.01 | 3.46 ± 0.02 | 3.96 ± 0.02 | 8.97 ± 0.02 | 10.67 ± 0.02 | 12.51 ± 0.02 | 12.82 ± 0.02 | 12.59 ± 0.02 |
| noPIP2 | 1.54 ± 0.02 | | 4.10 ± 0.03 | 9.42 ± 0.02 | 13.06 ± 0.02 | 13.96 ± 0.02 | 15.04 ± 0.02 | 13.94 ± 0.02 |
| PC | 3.61 ± 0.05 | | | | | | | 37.13 ± 0.04 |
| PMsparse | 6.13 ± 0.04 | 16.11 ± 0.04 | 12.18 ± 0.05 | 21.33 ± 0.04 | 21.82 ± 0.04 | 22.83 ± 0.05 | 24.63 ± 0.04 | 23.13 ± 0.05 |
| PMnoprot | | 24.58 ± 0.13 | 17.22 ± 0.12 | 28.66 ± 0.11 | 28.53 ± 0.13 | 29.31 ± 0.16 | 32.61 ± 0.13 | 29.56 ± 0.12 |
| PCnoprot | | | | | | | | 79.02 ± 0.30 |
| *Small Systems* | | | | | | | | |
| PM_s | 0.43 ± 0.00 | 2.24 ± 0.01 | 2.87 ± 0.02 | 7.71 ± 0.03 | 9.58 ± 0.03 | 11.67 ± 0.02 | 12.10 ± 0.02 | 11.63 ± 0.01 |
| noPIP2_s | 0.32 ± 0.02 | | 2.55 ± 0.04 | 8.22 ± 0.04 | 11.79 ± 0.01 | 12.51 ± 0.02 | 13.66 ± 0.02 | 12.55 ± 0.01 |
| noG_s | 0.56 ± 0.01 | 2.81 ± 0.02 | | 9.15 ± 0.03 | 10.82 ± 0.02 | 14.72 ± 0.03 | 14.41 ± 0.02 | 14.18 ± 0.02 |
| noPIP2G_s | 0.55 ± 0.0 | | | 8.83 ± 0.02 | 13.29 ± 0.0 | 16.55 ± 0.02 | 16.24 ± 0.02 | 15.44 ± 0.03 |
| noCh_s | 1.13 ± 0.02 | 6.13 ± 0.03 | 6.88 ± 0.03 | 20.35 ± 0.02 | 23.74 ± 0.04 | 29.02 ± 0.04 | | 28.10 ± 0.03 |
| noP2GCh_s | 1.47 ± 0.02 | | | 22.35 ± 0.01 | 30.09 ± 0.02 | 35.40 ± 0.03 | | 32.92 ± 0.02 |
| PC_s | 2.10 ± 0.06 | | | | | | | 36.24 ± 0.04 |
